# Protein Targets of Frankincense: A Reverse Docking Analysis of Terpenoids from *Boswellia* Oleo-Gum Resins

**DOI:** 10.3390/medicines5030096

**Published:** 2018-08-31

**Authors:** Kendall G. Byler, William N. Setzer

**Affiliations:** 1 Department of Chemistry, University of Alabama in Huntsville, Huntsville, AL 35899, USA; kendall.byler@uah.edu; 2 Aromatic Plant Research Center, 230 N 1200 E, Suite 102, Lehi, UT 84043, USA

**Keywords:** frankincense, *Boswellia*, cembranoids, cneorubenoids, boswellic acids, molecular docking

## Abstract

**Background:** Frankincense, the oleo-gum resin of *Boswellia* trees, has been used in traditional medicine since ancient times. Frankincense has been used to treat wounds and skin infections, inflammatory diseases, dementia, and various other conditions. However, in many cases, the biomolecular targets for frankincense components are not well established. **Methods:** In this work, we have carried out a reverse docking study of *Boswellia* diterpenoids and triterpenoids with a library of 16034 potential druggable target proteins. **Results:** *Boswellia* diterpenoids showed selective docking to acetylcholinesterase, several bacterial target proteins, and HIV-1 reverse transcriptase. *Boswellia* triterpenoids targeted the cancer-relevant proteins (poly(ADP-ribose) polymerase-1, tankyrase, and folate receptor β), inflammation-relevant proteins (phospholipase A2, epoxide hydrolase, and fibroblast collagenase), and the diabetes target 11β-hydroxysteroid dehydrogenase. **Conclusions:** The preferential docking of *Boswellia* terpenoids is consistent with the traditional uses and the established biological activities of frankincense.

## 1. Introduction

The genus *Boswellia* (Burseraceae) is made up of resiniferous trees and shrubs that are distributed across India, the Arabian peninsula, and Africa [1,2]. The genus is known for its aromatic terpenoid oleo-gum resin, frankincense. Frankincense has been a part of human religious ceremonies and ethnobotany for thousands of years [3]. Important frankincense-producing species include *B. carteri*, which grows in Somaliland and Puntland [1], *B. sacra*, found in Yemen, southern Oman, Somalia, and Somaliland [2], *B. frereana*, which is endemic to Somalia [2], *B. papyrifera*, primarily found in Sudan, Eritrea, and Ethiopia [4], and *B. serrata*, which grows primarily in India [5].

**Figure 1:**
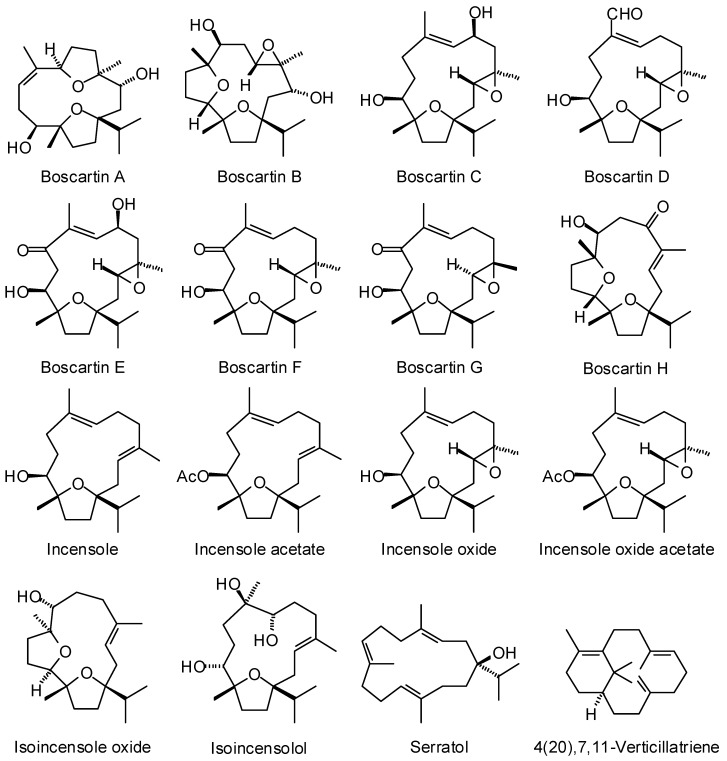
Macrocyclic diterpenoids found in *Boswellia* species.

**Figure 2:**
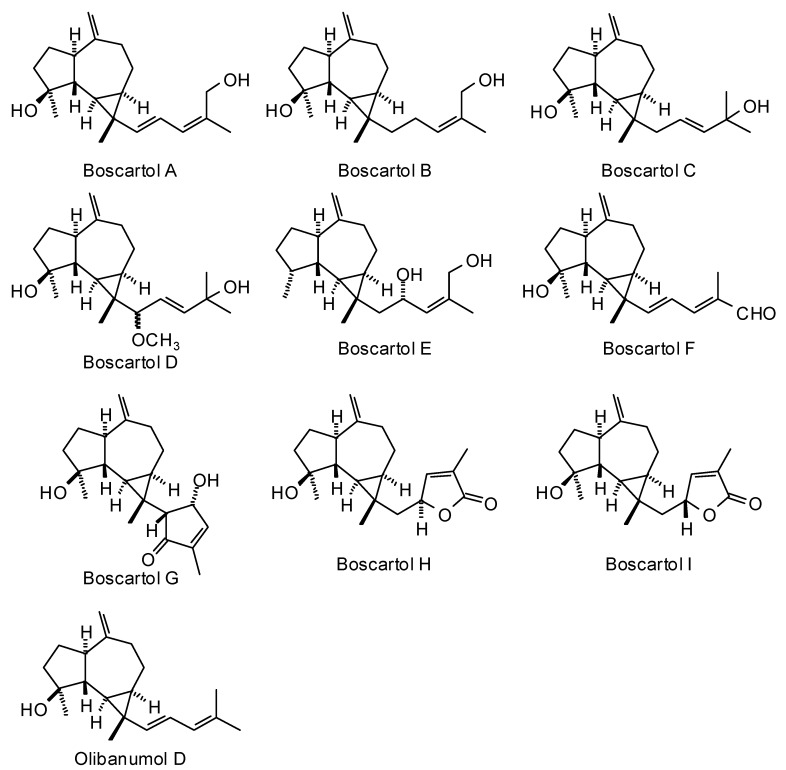
Cneorubenoid diterpenoids isolated from *Boswellia carteri*.

**Figure 3:**
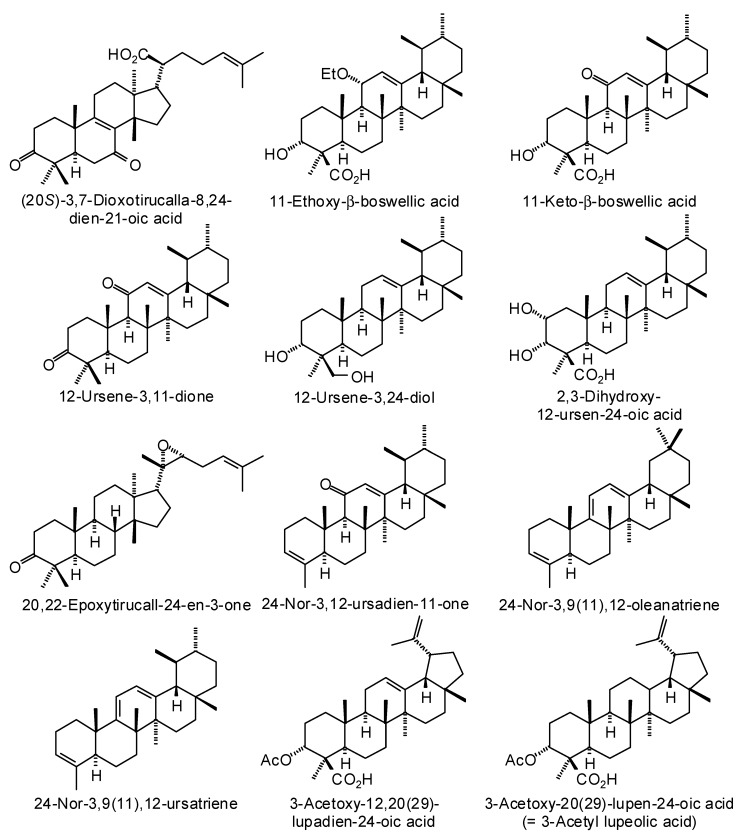

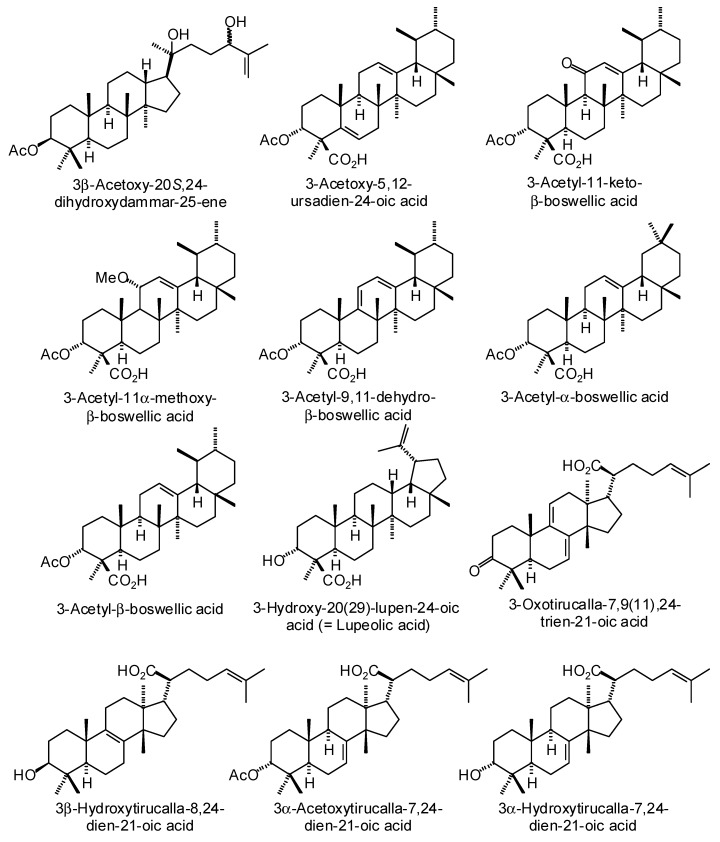

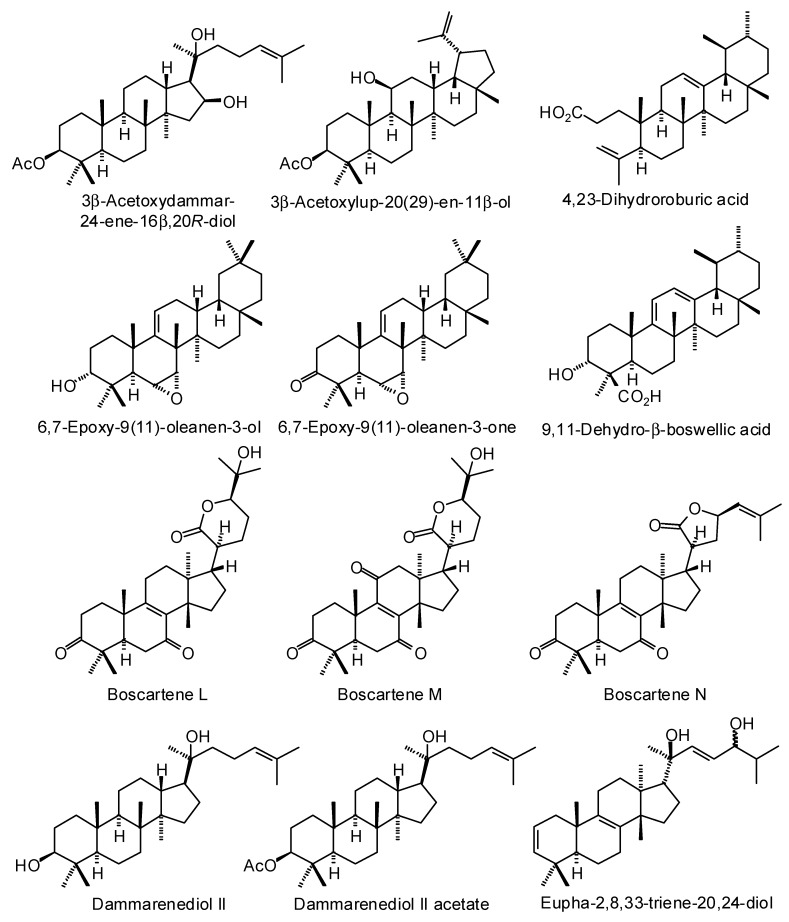

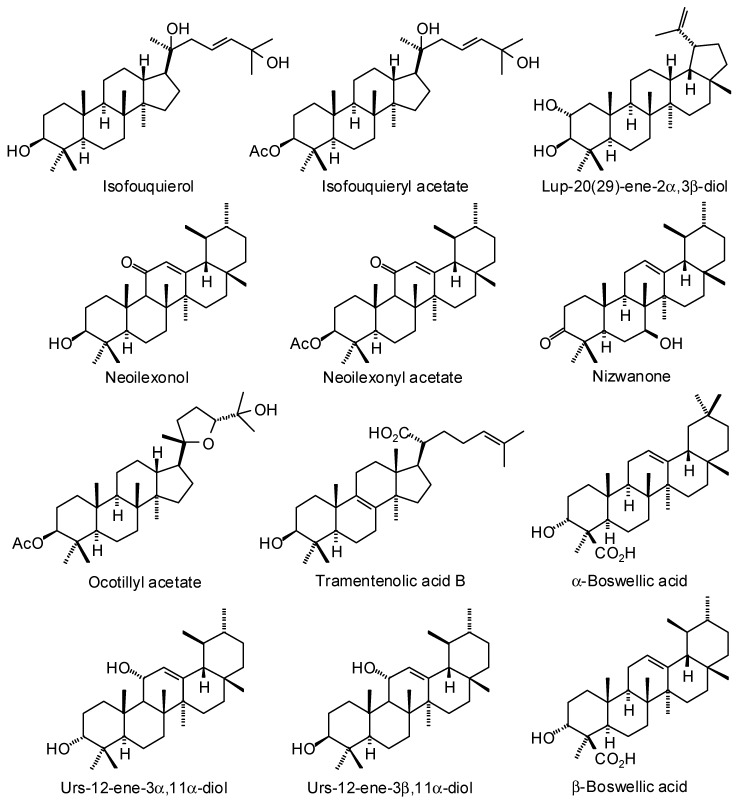

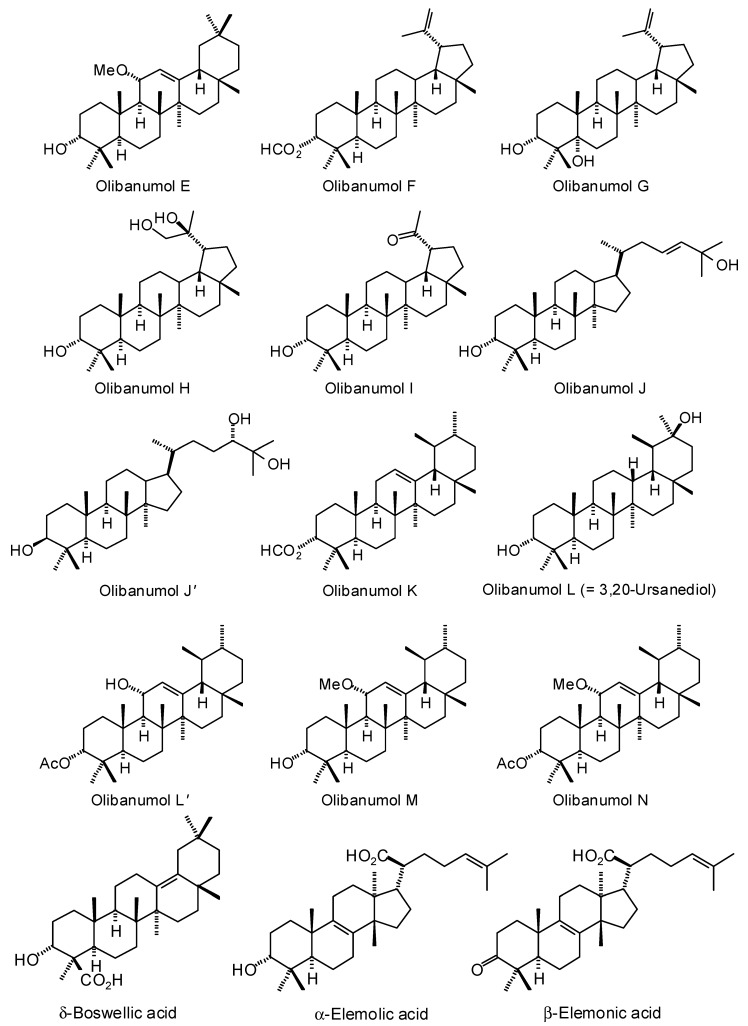
Triterpenoids isolated from *Boswellia* species.

Frankincense oleo-gum resin has been used traditionally to treat wounds [6], to treat inflammatory diseases [7], for oral hygiene [8], as well as for its psychoactive effects [9,10]. The biological activities of frankincense have been attributed to its essential oils [11] and its non-volatile diterpenoids and triterpenoids [6]. Although frankincense has been used for various maladies and conditions, and numerous biological activities have been attributed to frankincense, the particular biological targets are not well established. In this work, we have carried out a reverse molecular docking study of *Boswellia* cembranoid diterpenoids (Figure 1), cneorubenoid diterpenoids (Figure 2), and triterpenoids (Figure 3) against a library of 16,034 potential druggable target proteins.

The cembranoids incensole and incensole acetate were detected in the oleo-gum resin of *B. papyrifera*, while serratol was found in *B. carteri*, *B. sacra*, and *B. serrata* [12]. The boscartins have been isolated from the oleo-gum resin of *B. carteri* [13]. Incensole oxide has been isolated from *B. carteri* and the X-ray crystal structure determined [13,14]; both incensole oxide and incensole oxide acetate have been detected in small concentrations in the essential oil from the resin of *B. papyrifera* [15]. Isoincensole oxide [16,17] and isoincensolol [17] were isolated from *B. carteri* resin. Verticilla-4(20),7,11-triene and serratol and were isolated from *B. carteri* [18] and *B. serrata* [19], respectively.

*Boswellia carteri* oleo-gum resin is the source of several prenylated aromadendrane (cneorubenoid) diterpenoids (Figure 2) [20,21].

Numerous ursane, oleanane, lupane, dammarane, and tirucallane triterpenoids have been isolated and characterized from *Boswellia* species (Figure 3) [22]. *Boswellia serrata* has yielded α-boswellic acid, β-boswellic acid, 3-acetyl-α-boswellic acid, 3-acetyl-β-boswellic acid, 11-keto-β-boswellic acid, and 3-acetyl-11-keto-β-boswellic acid [23]. *Boswellia carteri* has yielded the oleanane triterpenoids α-boswellic acid, and 3-acetyl-α-boswellic acid; the ursane triterpenoids β-boswellic acid, 3-acetyl-β-boswellic acid, 11-keto-β-boswellic acid, 3-acetyl-11-keto-β-boswellic acid, 3-acetyl-11α-methoxy-β-boswellic acid, 9,11-dehydro-β-boswellic acid, and 3-acetyl-9,11-dehydro-β-boswellic acid; the lupane triterpenoids lupeolic acid and 3-acetyl lupeolic acid; and the tirucallane triterpenoids α-elemolic acid, β-elemonic acid, 3α-hydroxytirucalla-7,24-dien-21-oic acid, 3α-acetoxytirucalla-7,24-dien-21-oic acid, and 3β-hydroxytirucalla-7,24-dien-21-oic acid [24]. Olibanumols A, B, C, H, I, J’ [25], E, F, G [20], K, L’, M, and N [26] have been isolated from the oleo-gum resin of *B. carteri*. *B. carteri* resin has also yielded boscartenes L, M, and N, as well as trametenolic acid B, 3-oxotirucalla-7,9(11),24-trien-21-oic acid, and (20*S*)-3,7-dioxo-tirucalla-8,24,-dien-21-oic acid [27].

## 2. Materials and Methods

### 2.1. Ligand Preparation

Each ligand structure was prepared using Spartan’16 v. 2.0.7 (Wavefunction, Inc., Irvine, CA, USA). The lowest-energy conformations of the ligands were determined using the Merck Molecular Force Field (MMFF) [28]. In the case of the cembranoid macrocyclic ligands, further conformational analysis was carried out using density functional theory at the M06-2X/6-31G* level [29] with SM8 [30] aqueous solvent model [31].

### 2.2. Reverse Molelcular Docking

A reverse molecular docking study was carried out on each of the *Bosellia* terpenoids with the sc-PDB database of druggable binding sites [32]. Each compound was examined against the 16034 protein targets contained in the sc-PDB database. Prior to docking, all solvent molecules were removed from the protein structures. Co-crystallized enzyme cofactors were retained as cofactors and co-crystallized substrates or inhibitors were retained as ligands. Molecular docking was carried out using Molegro Virtual Docker v. 6.0.1 (Molegro ApS, Aarhus, Denmark) [33] as previously reported [34]. A python script was written to generate the Molegro input files; the jobs were run as a batch from the mvd.exe command line executable. The script took the co-crystallized ligands in each protein and wrote an input file that defined the search space for that docking as a sphere centered on the ligand’s center of mass. A 15-Å radius sphere was centered on the binding sites of each protein structure in order to permit each ligand to search. Standard protonation states of each protein, based on neutral pH, were used and charges were assigned based on standard templates as part of the Molegro Virtual Docker program. Each protein was used as a rigid model without protein relaxation. Flexible-ligand models were used in the docking optimizations. Different orientations of the ligands were search and ranked based on their “rerank” energy scores. A total of 100 runs for each ligand were carried out.

### 2.3. Conformational Analysis of Boscartol D

All calculations were carried out using Spartan’16 for Windows (Wavefunction, Inc., Irvine, CA, USA). Conformational profiles were carried out using molecular mechanics with the MMFF force field. Conformations with relative energies <20 kJ/mol were re-evaluated, with geometry optimization, using density functional theory (DFT, M06-2X/6-31G*) with a nonpolar solvent (CHCl_3_) model.

## 3. Results and Discussion

### 3.1. Cembranoid Diterpenoids

The macrocyclic cembranoid diterpenoids examined in this study are shown in Figure 1. The top-binding protein targets for each of the *Boswellia* cembranoids are summarized in Table 1. Included in Table 1 are the median docking energies for comparison. The top binding proteins for boscartin A were acetylcholinesterase (AChE) enzymes, *Torpedo californica* (TcAChE) and human (HsAChE). Boscartin B docked preferentially with human *N*-acetylgalactosaminyltransferase (HsGTA) as well as with the bacterial targets *Serratia marcescens* chitinase B (SmChiB), *Helicobacter pylori* peptide deformylase (HpPDF), and *Mycobacterium tuberculosis* 7,8-diaminopelargonic acid synthase (MtBioA). The proteins with the most exothermic docking for boscartin C were *Escherichia coli* aspartate transaminase (EcAspTA), murine acetylcholinesterase (MmAChE), and *Daboia russelii* (Russell’s viper) phospholipase A_2_ (DrPLA2). Boscartin D showed excellent docking with TcAChE (PDB 2cek, *E*_dock_ = −115.3 kJ/mol) and EcAspTA. The best protein targets for boscartin E were TcAChE, HpPDF, and MtBioA. Boscartin F showed preferential docking energies with human pyruvate kinase M2 (HsPKM2), TcAChE, and HpPDF. Boscartin G showed excellent docking properties with acetylcholinesterases TcAChE and HsAChE. The proteins with the most exothermic docking energies with boscartin H were human *N*-acetylgalactosaminyltransferase (HsGTA), DrPLA2, SmChiB, and MmAChE.

Incensole docked well with TcAChE, human aldo-keto reductase 1C3 (HsAKR1C3), and SmChiB. Incensole acetate preferentially targeted bacterial proteins EcAspTA, HpPDF, and *Burkholderia cepacia* phenazine biosynthesis protein A/B (BcPhzA/B). The preferred protein targets for incensole oxide were EcAspTA, human immunodeficiency virus type 1 reverse transcriptase (HIV-1-RT), TcAChE, BcPhzA/B, and MtBioA. Incensole oxide acetate gave excellent docking energies to EcAspTA, TcAChE, MmAChE, and HIV-1-RT. Isoincensole oxide docked well to BcPzhA/B and TcAChE. The protein targets that showed the best docking energies with isoincensolol were DrPLA2, *Staphylococcus aureus* multidrug binding protein QacR (SaQacR), and human dehydroepiandrosterone sulfotransferase (HsSULT2A1).

Every cembranoid ligand showed excellent docking properties to acetylcholinesterases (Table 2). Acetylcholinesterase has been identified as a target for treatment of Alzheimer’s disease [35]. This is notable because frankincense (*Boswellia* spp.) resins have been used in Persian traditional medicine as an anti-Alzheimer’s agent [36,37]. Animal models (rat) of Alzheimer’s disease [38,39,40] and human clinical trials [41,42] showed beneficial effects on memory with frankincense.

**Figure 4:**
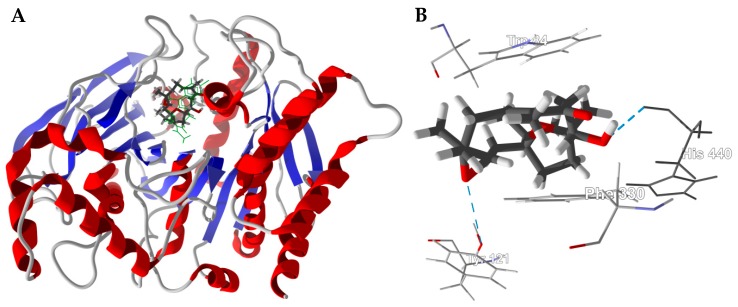
Lowest-energy docked pose of boscartin G with *Torpedo californica* acetylcholinesterase (TcAChE, PDB 1e66). (**A**): Ribbon structure of TcAChE with boscartin G in the active site; the co-crystallized ligand (huprene X) is shown as a green wire figure. (**B**): Key interactions of boscartin G with amino acids in the active site of TcAChE; hydrogen bonds are shown as blue dashed lines.

The lowest-energy docked pose of boscartin G with TcAChE (PDB 1e66, Figure 4A) shows the ligand to adopt the lowest-energy conformation as calculated by density functional theory at the M06-2X/6-31G*/SM8 level [31]. Key interactions between boscartin G and TcAChE are hydrophobic interactions between the ligand and aromatic amino acid side chains of Trp84, Phe330, and His440 (Figure 4B). In addition, there are hydrogen-bonding interactions between the oxirane ring of the ligand and the phenolic -OH of Tyr121 and the C(11)-OH of the ligand and the peptide C=O of His440 (Figure 4B).

**Figure 5:**
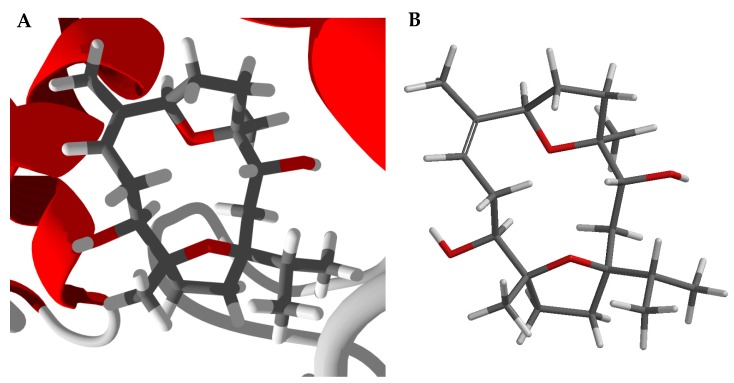
Boscartin A. (**A**): Lowest-energy docked pose of boscartin A with *Torpedo californica* acetylcholinesterase (PDB 2cek). (**B**): Calculated lowest-energy conformation of boscartin A at the M06-2X/6-31G*/SM8 level of theory [31].

Boscartin A occupies the active site of TcAChE (Figure 5A, PDB 2cek). As observed for boscartin G with TcAChE, key interactions between the docked ligand and the protein are hydrophobic interactions with Trp84, Phe330, and His440, and a hydrogen-bond between the C(11)-OH of the ligand and the His440 peptide C=O. The conformation of the lowest energy docked pose of boscartin A (Figure 5A) is the same as the lowest-energy calculated (M06-2X/6-31G*/SM8, Figure 5B) [31] and not that found in the X-ray crystal structure [13].

A number of bacterial proteins were targeted by *Boswellia* cembranoids (Table 3). *Helicobacter pylori* peptide deformylase (HpPDF) and *Escherichia coli* aspartate transaminase (EcAspTA) were particularly well targeted, while boscartin C and E and incensole oxide acetate showed remarkably exothermic docking energies. *Boswellia* resin extracts have shown in-vitro antibacterial activity [43,44,45], and frankincense resins have been used traditionally to treat wounds [6,46] and for oral hygiene [8]. Furthermore, *B. papyrifera* resin has shown activity against methicillin-resistant *Staphylococcus aureus* (MRSA) [47] and *B. serrata* resin showed activity in a clinical trial against plaque-induced gingivitis [48]. The selective targeting of bacterial proteins by *Boswellia* cembranoids corroborates the traditional medicinal uses and the demonstrated antibacterial activities of frankincense.

The potent docking properties of *Boswellia* cembranoids with HpPDF are particularly noteworthy. There is a strong association between colonization of the human stomach by *Helicobacter pylori* and gastrointestinal illnesses such as chronic gastritis and peptic ulcers [49]. Frankincense has been used traditionally to treat stomach disturbances [4] and ulcers [46]. In addition, *Boswellia* extracts have been shown in clinical studies to be helpful in treating ulcerative colitis [50].

**Figure 6:**
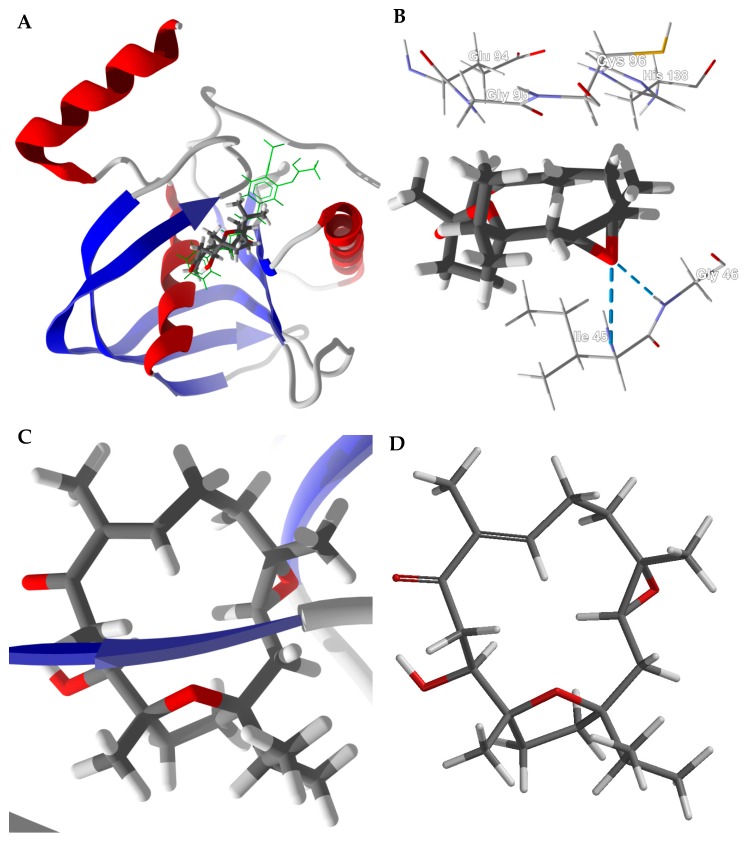
Docking of boscartin G with *Helicobacter pylori* peptide deformylase (HpPDF, PDB 2cek). (**A**): Ribbon structure of HpPDF showing boscartin G in the active site; the co-crystallized ligand, 4-{(1*E*)-3-oxo-3-[(2-phenylethyl)amino]prop-1-en-1-yl}-1,2-phenylene diacetate, is shown as a green stick figure. (**B**): Key interactions of boscartin G in the active site of HpPDF; hydrogen-bonds are shown as blue dashed lines. (**C**): Conformation of boscartin G docked to HpPDF. (**D**): Lowest-energy conformation of boscartin G determined by density functional calculations (M06-2X/6-31G*/SM8) [31].

Boscartin G is the strongest binding *Boswellia* cembranoid ligand with HpPDF (PDB 2ew5). The lowest-energy docked pose is shown in Figure 6. Boscartin G occupies the active site of HpPDF at the same location as the co-crystallized ligand, 4-{(1*E*)-3-oxo-3-[(2-phenylethyl)amino]- prop-1-en-1-yl}-1,2-phenylene diacetate, a cavity surrounded by Ile45, Gly95, Glu94, His138, Cys96, and Gly46 (Figure 6B). The ligand forms two hydrogen-bonds with the peptide N-H groups of Ile45 and Gly46. The docked structure of boscartin G with HpPDF shows the same conformation (Figure 6C) as that predicted from DFT calculations (Figure 6D) [31].

**Figure 7:**
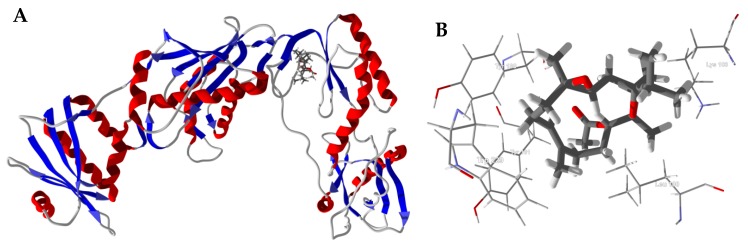

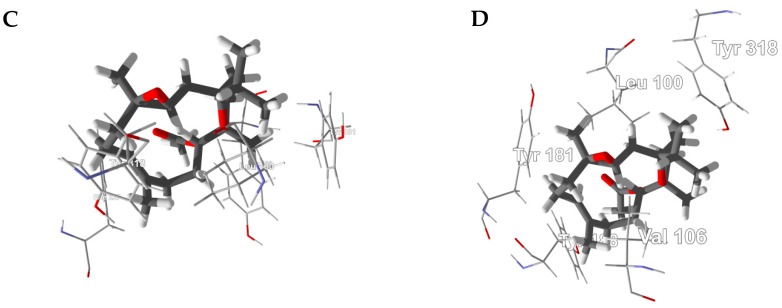
Molecular docking of incensole oxide acetate with HIV-1 reverse transcriptase. (**A**): Ribbon structure of HIV1-RT (PDB 3mee) showing incensole oxide acetate in the active site. (**B**): Key interactions of incensole oxide acetate in the active site of HIV1-RT (PDB 3mee). (**C**): Key interactions of incensole oxide acetate in the active site of HIV1-RT (PDB 3lal). (**D**): Key interactions of incensole oxide acetate in the active site of HIV1-RT (PDB 3t19).

Several *Boswellia* cembranoids showed selective docking to HIV-1 reverse transcriptase (HIV1-RT) (Table 4). In particular, incensole oxide acetate showed excellent docking (*E*_dock_ < −100 kJ/mol) to four of the seven HIV1-RT protein crystal structures. The lowest-energy docked pose of incensole oxide acetate with HIV-1 reverse transcriptase (PDB 3mee) is shown in Figure 7. Key interactions between the ligand and the protein are Tyr181, Tyr188, Leu100, Trp229, and Lys103 (Figure 7B). Interestingly, the docking energies for the cembranoids to PDB 3lal and 3t19 are, on average, lower than for the other protein structures. The differences in docking energies can be attributed to the arrangements of the amino acid residues at the binding sites, resulting in different orientations of the docked ligands. Thus, for example, the key amino acids interacting with incensole oxide acetate in PDB 3lal are Tyr188, Leu100, Tyr181, Phe227, and Tyr318 (Figure 7C), while PDB 3t19 had Leu100, Tyr188, Val106, Tyr318, and Tyr181 (Figure 7D). That is, binding sites of the protein crystal structures are heavily influenced by the co-crystallized ligands. Both methanol and aqueous extracts of *Boswellia carteri* have demonstrated HIV-1 reverse transcriptase activity [51].

### 3.2. Cneorubenoid Diterpenoids

The cneorubenoid diterpenoids, boscartols A–I and olibanumol D, can be considered to be prenylated aromadendranes (Figure 2), and have been isolated from the oleo-gum resin of *Boswellia carteri* [21]. The absolute configuration of the C(15) of boscartol D was not experimentally determined [21]. Nevertheless, both diastereomers, (15*R*)-boscartol D and (15*S*)-boscartol D were used in the reverse docking. In addition, the stereochemistry of C(15) was determined theoretically using density functional theory (DFT) conformational analysis carried out at the M06-2X/6-31G* level of theory, including a non-polar (CHCl_3_) solvent model. A complete conformational analysis of (15*R*)-boscartol D was carried out giving 20 low-energy conformations (*E*_rel_ < 14.0 kJ/mol, accounting for 100% of the Boltzmann distribution of conformers). Similarly, conformational analysis of (15*S*)-boscartol D returned 13 low-energy (*E*_rel_ < 13.0 kJ/mol). For each of the conformations, the H-C(15)-C(16)-H dihedral angle was determined and the corresponding vicinal coupling constants (^3^*J*_HH_) calculated using both the original Karplus equation [52] and the Haasnoot/Altona generalized Karplus equation that includes correction terms for the electronegativity of substituents [53]. Accounting for the Boltzmann distribution, (15*R*)-boscartin D is predicted to have ^3^*J*_HH_ of 4.3 and 5.1 Hz, respectively. The (15*S*)-diastereomer, on the other hand, is calculated to have ^3^*J*_HH_ of 6.3 and 6.5 Hz, respectively. The reported ^3^*J*_HH_ coupling constant was 7.6 Hz [21]. Based on the calculated ^3^*J*_HH_ coupling constants, the stereochemistry of boscartol D is predicted to be (15*S*).

The protein targets that showed the best docking properties with *Boswellia* cneorubenoids are listed in Table 5, along with median docking energies. The protein that was best targeted by *Boswellia* cneorubenoids was *Bacillus anthracis* nucleotide adenylyltransferase (BaNadD, PDB 3hfj) with seven of the 11 ligands showing docking energies <−120 kJ/mol. Human folate receptor β (HsFRβ, PDB 4kn0 and 4kn1) was also well targeted with 7/11 cneorubenoids with *E*_dock_ < −120 kJ/mol. The strongest docking ligands were boscartol E and boscartol I, and both of these ligands targeted BaNadD (PDB 3 hfj) and HsFRβ (PDB 4kn0) very well.

Nicotinate mononucleotide adenylyltransferase (NadD) has been identified as a target for development of antibacterial agents. The excellent docking of cneorubenoids with BaNadD, along with the known antibacterial activity of frankincense [43,44,45], corroborates the traditional uses of frankincense to treat wounds [6,46].

**Figure 8:**
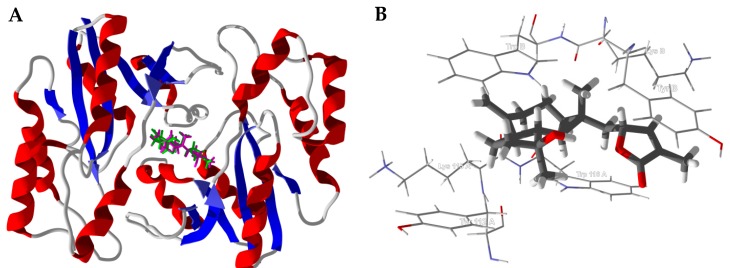
*Bacillus anthracis* nucleotide adenylyltransferase (BaNadD, PDB 3hfj). (**A**): Lowest-energy docked poses of boscartol E (magenta) and boscartol I (green) in the active site of the dimeric enzyme. (**B**): Boscartol I in the hydrophobic pocket formed at the interface of the two protein monomers.

*Bacillus anthracis* NadD (PDB 3hfj) is a dimeric structure with the active site at the interface of the two protein monomers (Figure 8). The active site is a hydrophobic pocket formed by Trp116A, Trp116B, Tyr112A, Tyr112B, Lys115A, and Lys115B (Figure 8B).

Human folate receptor β (HsFRβ) is overexpressed in activated macrophages associated with pathogenesis of inflammatory and autoimmune diseases [54] as well as neoplastic tissues [55]. Thus, antifolates that target folate receptors could be useful for the treatment of cancer and inflammatory diseases [56]. Clinical trials have demonstrated the encouraging results of frankincense treatment for inflammatory and autoimmune diseases such as rheumatoid arthritis, osteoarthritis, Crohn’s disease, and collagenous colitis [57]. *Boswellia* cneorubenoids may be playing a role in the anti-inflammatory activity of frankincense.

**Figure 9:**
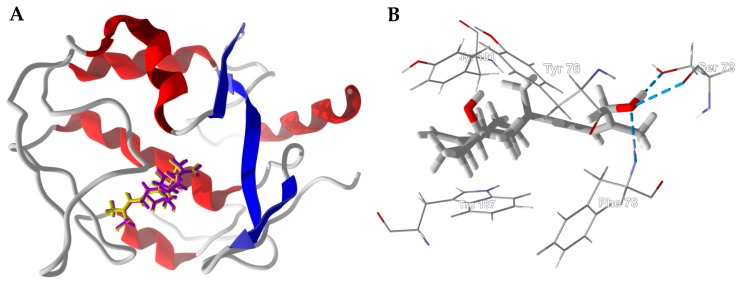
Human folate receptor β (HsFRβ, PDB 4kn1). (**A**): Ribbon structure of protein showed the docked poses of boscartin A (yellow) and boscartin B (purple). (**B**): Lowest-energy docked pose of boscartin A in the binding site of HsFRβ. Hydrogen-bonds are shown as blue dashed lines.

The boscartols docked with HsFRβ in the folate binding site (Figure 9). The cyclopropazulane ring is surrounded by aromatic amino acids Trp187, Tyr101, Tyr76, and Phe78 (Figure 9B). In the case of boscartol A and boscartol B, the terminal –OH group is held in place by hydrogen bonds to Ser73 and Phe78 (Figure 9B).

### 3.3. Boswellia Triterpenoids

The *Boswellia* triterpenoids examined in this reverse docking study are shown in Figure 3 and the target proteins with the best docking energies for each triterpenoid ligand are summarized in Table 6. The most receptive protein targets for *Boswellia* triterpenoids were *Staphylococcus aureus* multidrug binding protein (SaQacR, PDB 3bt9) with an average docking energy (*E*_dock_) of −111.9 kJ/mol and human fibroblast collagenase (PDB 1cgl) with an average docking energy (*E*_dock_) of −110.5 kJ/mol.

The best overall triterpenoid ligand-protein target pairs were boscartene N with human tankyrase 2 (HsTANK2, PDB 3ua9), *E*_dock_ = −157.2 kJ/mol; 3α-hydroxytirucalla-7,24-dien-21-oic acid with HsTANK2, *E*_dock_ = −154.3 kJ/mol; α-elemolic acid with HsTANK2, *E*_dock_ = −152.7 kJ/mol; 3-oxotirucalla-7,9(11),24-trien-21-oic acid with HsTANK2, *E*_dock_ = −151.0 kJ/mol; 3α-acetoxytirucalla-7,24-dien-21-oic acid with human glucokinase (PDB 4ixc), *E*_dock_ = −150.4 kJ/mol; isofouquieryl acetate with Guinea pig (*Cavia porcellus*) 11β-hydroxysteroid dehydrogenase type 1 (11βHSD1, PDB 3lz6), *E*_dock_ = −150.1 kJ/mol; 3β-acetoxy-20*S*,24*S*-dihydroxydammar-25-ene with *Enterobacter cloacae* pentaerythritol tetranitrate reductase (EcPETNR, PDB 2aba), *E*_dock_ = −149.9 kJ/mol; and 3β-acetoxydammar-24-ene-16β,20*R*-diol with Guinea pig 11β-hydroxysteroid dehydrogenase type 1 (Cp11βHSD1, PDB 3lz6), *E*_dock_ = −149.6 kJ/mol.

Frankincense oleo-gum resins have been used in traditional medicine to treat a variety of inflammatory conditions, including arthritis, colitis, and asthma [58,59]. Boswellic acids, including β-boswellic acid, 11-keto-β-boswellic acid, and acetyl-11-keto-β-boswellic acid, have been implicated in the anti-inflammatory properties of *Boswellia* resins; these triterpenoid components are involved in inhibition of 5-lipoxygenase (5-LOX), inducible nitric oxide synthase (iNOS), cyclooxygenase-1 (COX-1) and cyclooxygenase-2 (COX-2) [60].

In-silico screening of the *Boswellia* triterpenoids was carried out against molecular targets of inflammation, including human pancreatic secretory phospholipase A2 (HsPLA2), porcine pancreatic phospholipase A2 (SsPLA2), human phosphoinositide 3-kinase (HsPI3K), human interkeukin-1 receptor associated kinase 4 (HsIRAK4), human glutathione transferase omega 1 (HsGSTO1), human 5-lipoxygenase (Hs5-LOX), mouse inducible nitric oxide synthase (MmiNOS), ovine COX-1 (OaCOX-1), murine COX-2 (MmCOX-2), human fibroblast collagenase (matrix metalloproteinase-1, HsMMP-1), murine soluble epoxide hydrolase 2 (MmEPHX2), human endoplasmic reticulum aminopeptidase 2 (HsERAP2), human soluble epoxide hydrolase 2 (HsEPHX2), and human phosphodiesterase 4B (HsPDE4B). The docking energies of the triterpenoid ligands with inflammatory target proteins are shown in Table 7.

The *Boswellia* triterpenoids showed relatively weak docking to 5-LOX, iNOS, PI3K, IRAK4, or GSTO1, and no docking at all to either COX-1 or COX-2 (positive docking energies). However, several *Boswellia* triterpenoids showed relatively strong docking (*E*_dock_ < −120 kJ/mol) to human endoplasmic reticulum aminopeptidase 2 (HsERAP2), including 3β-acetoxy-20*S*,24*S*- dihydroxydammar-25-ene, 3β-acetoxydammar-24-ene-16β,20*R*-diol, isofouquieryl acetate, ocotillyl acetate, olibanumol J, and olibanumol J’. There is a significant association of ERAP2 with psoriatic arthritis [61], and notably, *Boswellia* triterpenoids have shown promise for the treatment of psoriasis [62]. Similarly, inhibitors of phosphodiesterase 4B have shown promise in the treatment of psoriasis and atopic dermatitis [63], and isofouquierol and olibanumol J’ showed good docking properties with HsPDE4B with docking energies of −128.3 and −128.8 kJ/mol, respectively. The only other strong docking observed was olibanumol J with murine soluble epoxide hydrolase 2 (MmEPHX2), 3α-acetoxytirucalla-7,24-dien-21-oic acid with human matrix metalloproteinase-1 (HsMMP-1), (20*S*)-3,7-dioxotirucalla-8,24-dien-21-oic acid with porcine pancreatic phospholipase A2 (SsPLA2), and 3β-acetoxy-20*S*,24-dihydroxydammar-25-ene with human pancreatic secretory phospholipase A2 (HsPLA2). The targeting of matrix metalloproteinase-1 (fibroblast collagenase) is noteworthy; *B. serrata* extract has shown clinical efficacy as a treatment for osteoarthritis of the knee [64].

Note that β-boswellic acid, 11-keto-β-boswellic acid, and 3-acetyl-11-keto-β-boswellic acid had relatively weak docking with inflammation-relevant protein targets. It may be that these *Boswellia* triterpenoids, rather than inhibiting particular enzyme targets, are inhibiting the secretion of pro-inflammatory cytokines such as tumor necrosis factor α (TNFα), interleukin 1 (IL-1), IL-6, IL-12, IL-18, or interferon γ (IFN-γ) [65].

**Figure 10:**
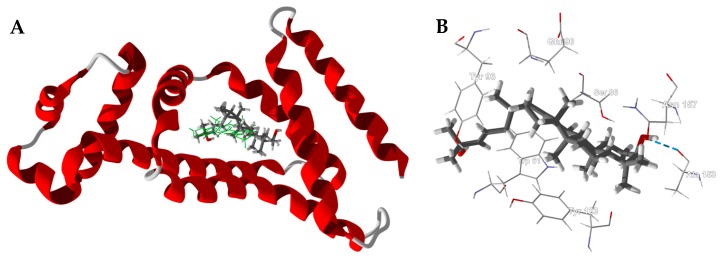
Lowest-energy docking pose of olibanumol J with *Staphylococcus aureus* multidrug binding protein (SaQacR, PDB 3bti). (**A**): Ribbon structure of SaQacR showing olibanumol J (*E*_dock_ = −132.6 kJ/mol) in the active site. The co-crystallized ligand, berberine (*E*_dock_ = −113.8 kJ/mol), is shown as a green wire structure. (**B**): Key interactions of olibanumol J in the active site of SaQacR. The hydrogen-bond is indicated by a blue dashed line.

Olibanumols G, H, I, and J all showed selective docking to *S. aureus* multidrug binding protein (SaQacR). The olibanumols occupy the binding site of SaQacR, same site as the co-crystallized ligand in PDB 3bti (Figure 10). The site is made up of aromatic amino acids Trp61, Tyr93, and Tyr123, forming a hydrophobic pocket. The triterpenoid ligand olibanumol J also has a hydrogen-bonding interaction between C(3)-OH of the ligand and the amide C=O of Ala153. Other key interacting amino acids in the binding site are Ser86, Glu90, and Asn157. In addition to the olibanumols, six additional triterpenoids also showed selective docking to SaQacR, 3-acetoxy-12,20(29)-lupadien-24-oic acid, lupeolic acid, 3-oxotirucalla-7,9(11),24-trien-21-oic acid, 4,23-dihydroburic acid, 9,11-dehydro-β-boswellic acid, and boscartene N.

**Figure 11:**
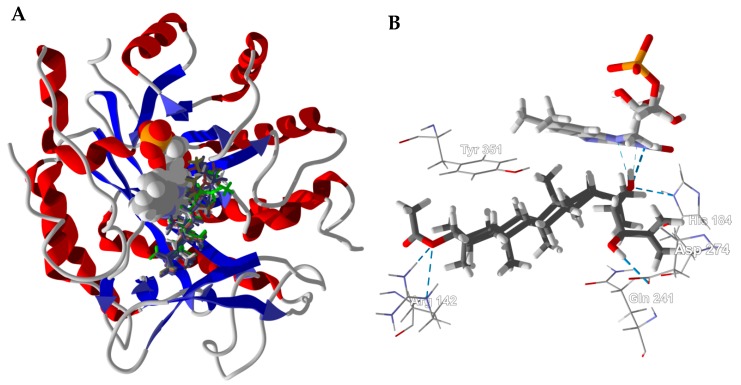
*Enterobacter cloacae* pentaerythritol tetranitrate reductase (EcPETNR, PDB 2aba). (**A**): Ribbon structure of EcPETNR with docked dammarane triterpenoids (stick figures). The riboflavin monophosphate cofactor is shown as a space-filling model. (**B**): Lowest-energy docked pose of 3β-acetoxy-20*S*,24*S*-dihydroxydammar-25-ene. Hydrogen-bonding interactions are indicated with blue dashed lines.

Another antibacterial target protein that showed good docking properties was *Enterobacter cloacae* pentaerythritol tetranitrate reductase (EcPETNR) with seven *Boswellia* dammarane triterpenoids showing selective docking to this target, 3β-acetoxy-20*S*,24-dihydroxydammar-25-ene (both diastereomers), 3β-acetoxydammar-24-ene-16β,20*R*-diol, dammarenediol II acetate, isofouquierol, isofouquieryl acetate, and ocotillyl acetate. These dammarane triterpenoids occupy the active site of EcPETNR, near to the riboflavin monophosphate redox cofactor, with very similar docked poses (Figure 11). Key intermolecular contacts are hydrogen bonding of the C(3) acetoxygroup with Arg142, hydrogen-bonding of C(20)-OH with the riboflavin monophosphate cofactor and His184 (Figure 11B). In the case of 3β-acetoxy-20*S*,24*S*-dihydroxydammar-25-ene, there is also a hydrogen-bond between the C(24)-OH group and Asp274.

**Figure 12:**
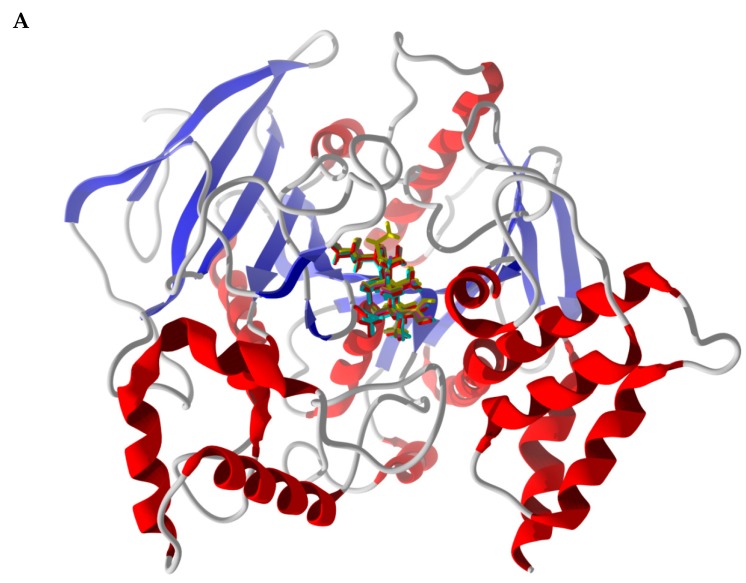

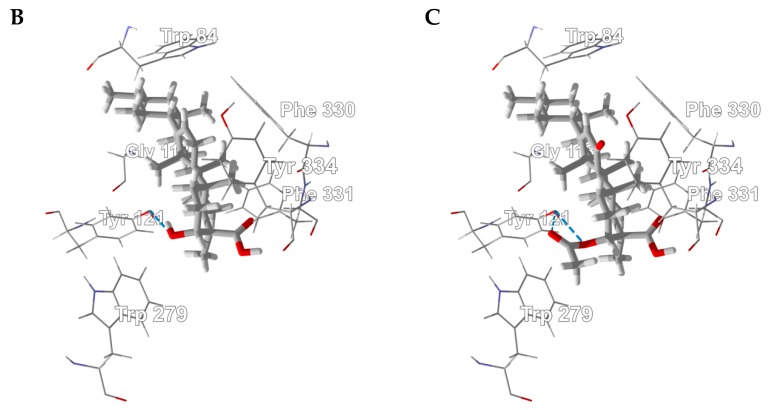
Lowest-energy docked poses of β-boswellic acid derivatives with *Torpedo californica* acetylcholinesterase (TcAChE, PDB 3i6m). (**A**): Ribbon structure with docked ligands, β-boswellic acid (brown), 11-keto-β-boswellic acid (magenta), 11-ethoxy-β-boswellic acid (yellow), 3-acetyl-β-boswellic acid (red), and 3-acetyl-11-keto-β-boswellic acid (aqua). (**B**): Molecular environment of docked β-boswellic acid in the active site of TcAChE. (**C**): Molecular environment of docked 3-acetyl-11-keto-β-boswellic acid in the active site of TcAChE.

Drugs that reversibly inhibit acetylcholinesterase are currently being explored to treat Alzheimer’s disease [66], and *Torpedo californica* and murine acetylcholinesterases have been used as model enzyme targets for anticholinesterase inhibition [67]. β-Boswellic acid and its derivatives (11-keto-β-boswellic acid, 11-ethoxy-β-boswellic acid, 3-acetyl-β-boswellic acid, and 3-acetyl-11-keto-β-boswellic acid) showed selective docking to *Torpedo californica* acetylcholinesterase (TcAChE, PDB 3i6m). Interestingly, increasing oxygenation resulted in more exothermic docking energies (−118.1, −120.8, −124.6, −128.3, and −129.7 kJ/mol, respectively). These β-boswellic acid derivatives all adopt the same lowest-energy poses in the active site of the enzyme (Figure 12). It is tempting to suggest that the ordering of docking energies is due to increasing hydrogen-bonding of the more oxygenated ligands. However, the only hydrogen bonding seen is with the C(3)-substituent of the ligand (either –OH or –OAc) with Tyr121 (see Figure 10B. Note that the active site of the acetylcholinesterase is surrounded by aromatic amino acids (Trp84, Tyr334, Tyr121, Phe330, Phe331, and Trp279). The more important interactions, therefore, are van der Waals hydrophobic interactions between the ligand and the aromatic amino acids. The trend in docking energies for these β-boswellic acid derivatives is likely due to the increased number of heavy atoms and increasing molecular weight. Olibanumols K, L’, M, and N also showed selective docking to acetylcholinesterase proteins. The strongest-docking triterpenoid ligands to TcAChE were 4,23-dihydroburic acid (*E*_dock_ = −135.8 kJ/mol) and 3-acetoxy-5,12-ursadien-24-oic acid (*E*_dock_ = −131.7 kJ/mol); olibanumol N docked strongly with MmAChE (*E*_dock_ = −133.0 kJ/mol). The excellent docking properties of *Boswellia* triterpenoid ligands to acetylcholinesterase supports the clinical use of frankincense to treat Alzeimer’s disease [41].

Several protein targets related to cancer were targeted by *Boswellia* triterpenoids, including poly(ADP-ribose) polymerase-1 (PARP-1), tankyrase-1 (TANK1), tankyrase-2 (TANK2), folate receptor β (FRβ), and histone deacetylase (HDAC). Poly(ADP-ribose) polymerase-1 (PARP-1) is an important enzyme for the repair of single-strand DNA breaks, and inhibition of PARP-1 can cause multiple double strands DNA breaks to occur, leading to cell death [68]. Several types of cancers are more dependent on PARP-1 than normal cells, so PARP-1 has become an attractive target for cancer chemotherapy [69,70]. At least 15 *Boswellia* triterpenoids showed selective docking to PARP-1 (see Table 6). The strongest-docking ligands for human PARP-1 were (20*S*)-3,7-dioxotirucalla-8,24-dien-21-oic acid (*E*_dock_ = −141.8 kJ/mol) and eupha-2,8,22-triene-20,24*R*-diol (*E*_dock_ = −139.8 kJ/mol).

Folate receptors are overexpressed in cancer cells, presumably due to the increased requirement of cancer cells for folic acid needed in cell proliferation [71] and folate receptor-β is overexpressed in lung, liver, skin, and soft tissue tumors, as well as associated stromal cells. Folate receptors, therefore, show promise as chemotherapeutic targets for cancer and other human pathologies [72]. Three of the *Boswellia* triterpenoids in this study showed excellent docking properties to human folate receptor β (HsFRβ), namely dammarenediol II acetate, isofouquierol, and isofouquieryl acetate, with docking energies of −132.3, −130.4, and −137.0 kJ/mol, respectively.

Histone deacetylases (HDAC) are enzymes that remove acetyl groups from lysine residues of histones, which allow the histones to envelope DNA more tightly. Thus, HDACs can affect cell growth and differentiation and cell death [73,74]. Histone deacetylase has been recognized as a promising target for cancer chemotherapy [75,76]. Reverse docking of *Boswellia* triterpenoids has revealed α-elemolic acid to preferentially dock to *Aquifex aeolicus* histone deacetylase (AaHDAC).

Frankincense-containing formulations had been used in ancient Greece for treating various malignant tumors [77]. Frankincense has been used in the Indian traditional medicine (Ayurveda) and in Traditional Chinese Medicine (TCM) as a treatment for proliferative diseases [78]. In addition, extracts of frankincense oleo-gum resins have shown in-vitro cytotoxic activity on several human tumor-derived cell lines [58,79,80,81], and these activities have been attributed to boswellic acids [82]. Interestingly, although β-boswellic acid [83] and 3-acetyl-11-keto-β-boswellic acid [84] have shown antineoplastic activities, this reverse-docking study did not reveal particularly notable docking properties to cancer-relevant protein targets. It may be that the boswellic acids and derivatives are targeting inflammatory pathways [85,86] or multiple targets as their mechanisms of antineoplastic activities [82,84].

**Figure 13:**
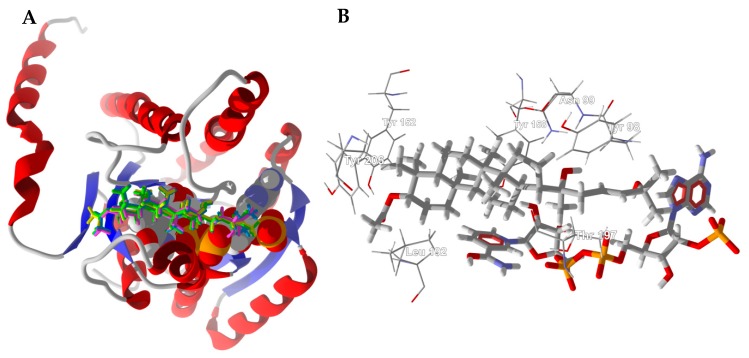
Guinea pig 11β-hydroxysteroid dehydrogenase type 1 (Cp11β-HSD, PDB 3lz6). (**A**): Lowest-energy docked poses of 3β-acetoxy-20*S*,24*R*-dihydroxydammar-25-ene (magenta), 3β-acetoxydammar-24-ene-16β,20*R*-diol (green), dammarenediol II acetate (yellow), and isofouquieryl acetate (blue) with Cp11β-HSD. The NADPH cofactor is shown as a space-filling model. (**B**): Isofouqueryl acetate in the active site of Cp11β-HSD showing the molecular environment.

Several *Boswellia* triterpenoids showed good docking properties to 11β-hydroxysteroid dehydrogenase type 1 (11βHSD1). For example, the dammarane triterpenoids 3β-acetoxy- 20*S*,24-dihydroxydammar-25-ene, 3β-acetoxydammar-24-ene-16β,20*R*-diol, dammarenediol II, dammarenediol II acetate, isofouquierol, and isofouquieryl acetate showed excellent docking energies with *Cavia porcellus* 11β-hydroxysteroid dehydrogenase type 1 (Cp11βHSD1, PDB 3lz6). These dammarane triterpenoids all occupy the same position in the active site of the enzyme, blocking access to the NADPH cofactor (Figure 13). The docked dammaranes are sandwiched between the NADPH cofactor and hydrophobic amino acids Tyr152, Tyr98, Tyr158, Leu192, and Tyr206 (Figure 13B). There are also close contacts, but no apparent hydrogen-bonds, with Thr197 and Asn99. 11β-Hydroxysteroid dehydrogenase type 1 mediates the interconversion of cortisone and cortisol and overexpression of 11βHSD1 can lead to metabolic disease, characterized by visceral obesity, hyperlipidemia, hypertension, glucose intolerance, insulin resistance, and type II diabetes [87,88]. Thus, inhibition of 11βHSD1 may serve as a treatment option for metabolic syndrome and type II diabetes [89,90].

*Boswellia serrata* is used traditionally by diabetic patients in Iran, and *B. serrata* supplementation has shown clinical benefit in blood lipid and glucose levels in type II diabetic patients [91,92]. Furthermore, *B. serrata* resin extract has been shown to prevent increase in blood glucose levels in streptozotocin-induced diabetic mice [93]. Similarly, *B. glabra* extracts have shown hypoglycemic effects in alloxan-induced diabetic rats [94]. The selective targeting of Guinea pig 11βHSD1 and human 11βHSD1 by *Boswellia* triterpenoids is consistent with the traditional use and anti-diabetic activities of *Boswellia* oleo-gum resin.

**Figure 14:**
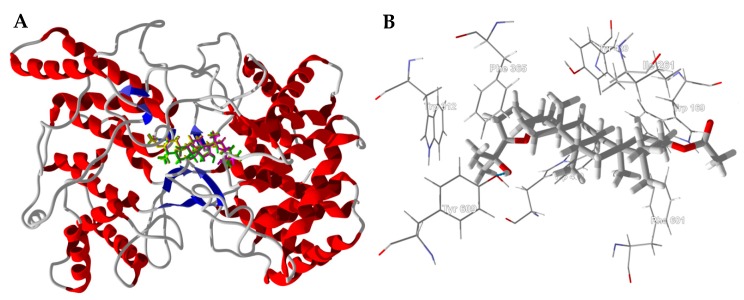
*Alicyclobacillus acidocaldarius* oxidosqualene cyclase (AaOSC, PDB 1h36). (**A**): Lowest energy docked poses of ocotillyl acetate (magenta), 3β-acetoxydammar-24-ene-16β,20*R*-diol (green), and dammarenediol II acetate (yellow) with AaOSC. (**B**): Molecular environment of docked ocotillyl acetate in the active site of AaOSC.

Oxidosqualene cyclases (OSCs) are enzymes that catalyze the cyclization of 2,3-epoxysqualene to form triterpenoids or steroids [95]. In mammals, cyclization of 2,3-epoxyaqualene leads to lanosterol, which can then be converted to cholesterol [96]. Inhibition of oxidosqualene cyclase, therefore, has emerged as a viable therapeutic option to treat hypercholesterolemia and atherosclerosis [97,98]. The oxidosqualene-hopene cyclase from the thermophilic bacterium *Alicyclobacillus acidocaldarius* is homologous to the human enzyme and has been crystallized with OSC inhibitors [99]. Several triterpenoid ligands, most notably ocotillyl acetate, 3β-acetoxydammar-24-ene-16β,20*R*-diol, and dammarenediol II acetate, docked well to AaOSC with docking energies of −144.6, −139.2, and −138.9 kJ/mol, respectively. These dammarane triterpenoids adopt the same positions in the active site of the enzyme (Figure 14). The active site of AaOSC is a hydrophobic pocket composed of Trp489, Trp169, Phe365, Ile261, Trp312, Phe601, and Tyr420. In addition, there is a hydrogen bond formed between the C(24)-OH of the ligand and the phenolic -OH of Tyr609. The structural similarities between triterpenoids and steroids are likely responsible for the docking properties of *Boswellia* triterpenoids to OSCs.

## 4. Conclusions

Numerous *Boswellia* terpenoid components have shown selective docking to bacterial protein targets, antineoplastic molecular targets, diabetes-relevant targets, protein targets involved in inflammatory disease conditions, and the Alzheimer’s disease target acetylcholinesterase. The molecular docking properties of *Boswellia* terpenoid components corroborate the traditional uses of frankincense, the clinical efficacy of frankincense, and the biological activities of *Boswellia* oleo-gum resins and components. Furthermore, the biomolecular targets identified in this work should lead to further exploration of development and improvement of inhibitors to treat these various disease states.

## Figures and Tables

**Table 1:** Protein targets with the most exothermic docking energies (*E*_dock_, kJ/mol) for *Boswellia* cembranoid ligands.

Ligand	PDB ^a^	*E*_dock_	Target Protein
Boscartin A	1e66	−119.7	*Torpedo californica* acetylcholinesterase (TcAChE)
	2cek	−123.7	*Torpedo californica* acetylcholinesterase (TcAChE)
	4bdt	−113.5	human acetylcholinesterase (HsAChE)
		−79.2	Median docking energy
Boscartin B	1h0g	−107.8	*Serratia marcescens* chitinase B (SmChiB)
	2ew5	−104.6	*Helicobacter pylori* peptide deformylase (HpPDF)
	3tfu	−107.5	*Mycobacterium tuberculosis* 7,8-diaminopelargonic acid synthase (MtBioA)
	3v0o	−110.4	human fucosylgalactoside α *N*-acetylgalactosaminyltransferase (HsGTA)
		−80.5	Median docking energy
Boscartin C	1ahg	−112.5	*Escherichia coli* aspartate aminotransferase (EcAspTA)
	1fv0	−108.2	*Daboia russelii* (Russell’s viper) phospholipase A_2_ (DrPLA2)
	1q83	−110.4	murine acetylcholinesterase (MmAChE)
	4g1n	−106.5	human pyruvate kinase isozyme M2 (HsPKM2)
		−91.2	Median docking energy
Boscartin D	1ahg	−114.4	*Escherichia coli* aspartate aminotransferase (EcAspTA)
	1xzq	−106.3	*Thermotoga maritima* GTP-binding protein TrmE (TmTrmE)
	2cek	−115.3	*Torpedo californica* acetylcholinesterase (TcAChE)
		−82.0	Median docking energy
Boscartin E	1ahg	−110.4	*Escherichia coli* aspartate aminotransferase (EcAspTA)
	1e66	−111.7	*Torpedo californica* acetylcholinesterase (TcAChE)
	2ew5	−107.5	*Helicobacter pylori* peptide deformylase (HpPDF)
		−68.4	Median docking energy
Boscartin F	2ew5	−109.5	*Helicobacter pylori* peptide deformylase (HpPDF)
	3i6m	−107.3	*Torpedo californica* acetylcholinesterase (TcAChE)
	4g1n	−108.5	human pyruvate kinase isozyme M2 (HsPKM2)
		−82.9	Median docking energy
Boscartin G	1e66	−126.4	*Torpedo californica* acetylcholinesterase (TcAChE)
	2cek	−116.8	*Torpedo californica* acetylcholinesterase (TcAChE)
	4bdt	−118.7	human acetylcholinesterase (HsAChE)
		−89.9	Median docking energy
Boscartin H	1fv0	−106.9	*Daboia russelii* (Russell’s viper) phospholipase A_2_ (DrPLA2)
	1w1t	−106.0	*Serratia marcescens* chitinase B (SmChiB)
	2gyw	−105.9	murine acetylcholinesterase (MmAChE)
	3v0o	−109.3	human fucosylgalactoside α *N*-acetylgalactosaminyltransferase (HsGTA)
		−88.4	Median docking energy
Incensole	2cek	−111.9	*Torpedo californica* acetylcholinesterase (TcAChE)
	3ugr	−109.7	human aldo-keto reductase 1C3 (HsAKR1C3)
	1h0g	−102.4	*Serratia marcescens* chitinase B (SmChiB)
		−78.1	Median docking energy
Incensole acetate	1ahg	−106.9	*Escherichia coli* aspartate aminotransferase (EcAspTA)
	3jun	−103.7	*Burkholderia cepacia* phenazine biosynthesis protein A/B (BcPhzA/B)
		−69.5	Median docking energy
Incensole oxide	1ahg	−108.6	*Escherichia coli* aspartate aminotransferase (EcAspTA)
	2cek	−103.1	*Torpedo californica* acetylcholinesterase (TcAChE)
	3jup	−103.5	*Burkholderia cepacia* phenazine biosynthesis protein A/B (BcPhzA/B)
	3mee	−106.7	HIV-1 reverse transcriptase (HIV-1 RT)
		−74.6	Median docking energy
Incensole oxide acetate	1ahg	−119.0	*Escherichia coli* aspartate aminotransferase (EcAspTA)
	1q83	−109.6	murine acetylcholinesterase (MmAChE)
	2cek	−110.4	*Torpedo californica* acetylcholinesterase (TcAChE)
	3i6m	−114.3	*Torpedo californica* acetylcholinesterase (TcAChE)
	3mee	−113.5	HIV-1 reverse transcriptase (HIV-1 RT)
		−91.9	Median docking energy
Isoincensole oxide	3jup	−103.3	*Burkholderia cepacia* phenazine biosynthesis protein A/B (BcPhzA/B)
		−80.5	Median docking energy
Isoincensolol	1fv0	−108.2	*Daboia russelii* (Russell’s viper) phospholipase A_2_ (DrPLA2)
	1jus	−107.9	*Staphylococcus aureus* multidrug binding protein (SaQacR)
	2qp4	−102.2	human dehydroepiandrosterone sulfotransferase (HsSULT2A1)
		−74.0	Median docking energy
Serratol	1e66	−104.1	*Torpedo californica* acetylcholinesterase (TcAChE)
	2cek	−106.1	*Torpedo californica* acetylcholinesterase (TcAChE)
	2ew5	−103.2	*Helicobacter pylori* peptide deformylase (HpPDF)
	4bdt	−103.0	human acetylcholinesterase (HsAChE)
		−78.1	Median docking energy
Verticillatriene	1w4l	−104.3	*Torpedo californica* acetylcholinesterase (TcAChE)
	3i6m	−100.8	*Torpedo californica* acetylcholinesterase (TcAChE)
	3i6z	−103.2	*Torpedo californica* acetylcholinesterase (TcAChE)
		−63.6	Median docking energy

**^a^** PDB: Protein Data Bank code.

**Table 2:** MolDock molecular docking energies (kJ/mol) of *Boswellia* cembranoids with acetylcholinesterase protein targets. ^a^

Ligand	TcAChE	TcAChE	TcAChE	TcAChE	TcAChE	TcAChE	MmAChE	MmAChE	HsAChE
	1e66	1h22	1w4l	2cek	3i6m	3i6z	1q83	2gyw	4bdt
Boscartin A	−119.7	−98.4	−99.5	−123.7	−98.2	−100.6	−95.9	−101.4	−113.5
Boscartin B	−95.1	−98.8	−92.5	−106.3	−99.2	−94.5	−94.3	−85.8	−106.3
Boscartin C	−87.7	−90.8	−94.7	−96.9	−104.1	−94.2	−110.4	−94.0	−86.6
Boscartin D	−99.7	−102.8	−93.1	−115.3	−103.7	−93.9	−92.5	−93.6	−90.2
Boscartin E	−111.7	−96.5	−94.5	−108.7	−95.5	−92.9	−86.0	−94.8	−90.6
Boscartin F	−93.2	−97.0	−98.5	−104.8	−107.3	−95.8	−101.1	−82.4	−77.3
Boscartin G	−126.4	−99.4	−94.5	−116.8	−101.5	−95.6	−111.1	−94.1	−118.7
Boscartin H	−100.1	−106.3	−103.6	−105.6	−103.0	−104.2	−98.8	−105.9	−97.8
Incensole	−89.7	−92.1	−89.5	−111.9	−94.1	−89.6	−90.3	−87.7	−53.4
Incensole acetate	−88.6	−97.2	−102.8	−102.5	−102.3	−101.7	−87.5	−85.0	−42.3
Incensole oxide	−89.6	−93.2	−92.7	−103.1	−96.9	−89.3	−101.9	−95.5	−93.5
Incensole oxide acetate	−109.8	−98.7	−96.8	−110.4	−114.3	−95.8	−109.6	−97.7	−102.6
Isoincensole oxide	−92.9	−98.9	−96.4	−103.6	−98.1	−89.9	−89.9	−90.3	−94.1
Isoincensolol	−88.5	−99.5	−92.4	−101.5	−98.3	−91.2	−77.0	−80.4	−73.6
Serratol	−104.1	−95.7	−92.2	−106.1	−94.5	−88.1	−96.4	−89.3	−104.0
Verticillatriene	−82.5	−91.2	−104.3	−85.3	−100.8	−103.2	−73.9	−98.1	−61.2

^a^ TcAChE = *Torpedo californica* acetylcholinesterase. MmAChE = *Mus musculus* (murine) acetylcholinesterase. HsAChE = human acetylcholinesterase.

**Table 3:** MolDock molecular docking energies (kJ/mol) of *Boswellia* cembranoids with bacterial target proteins. ^a^

Ligand	EcAspTA	SmChiB	SmChiB	SmChiB	SaQacR	SaQacR	SaQacR	SaQacR	HpPDF	BcPhzA/B	BcPhzA/B	MtBioA
	1ahg	1h0g	1w1t	3wd2	1jus	1rpw	3br2	3bti	2ew5	3jun	3jup	3tfu
Boscartin A	−116.9	−93.6	−99.4	−96.3	−99.4	−104.1	−94.9	−95.7	−101.8	−100.3	−107.8	−104.9
Boscartin B	−98.2	−107.8	−103.7	−91.5	−98.1	−106.5	−94.7	−96.5	−104.6	−98.4	−93.9	−107.5
Boscartin C	−112.5	−96.3	−97.3	−90.6	−100.0	−98.5	−98.4	−102.3	−106.2	−99.3	−106.4	−100.4
Boscartin D	−114.4	−99.7	−105.3	−91.5	−95.2	−106.2	−100.1	−96.3	−104.9	−98.0	−95.8	−99.3
Boscartin E	−110.4	−101.0	−105.9	−105.4	−99.2	−101.3	−95.3	−95.3	−107.5	−84.7	−96.7	−107.7
Boscartin F	−99.7	−97.3	−103.7	−89.9	−101.0	−95.7	−94.4	−85.2	−109.5	−87.7	−98.0	−95.1
Boscartin G	−105.5	−90.8	−93.0	−93.8	−95.1	−98.2	−92.2	−88.7	−112.7	−95.4	−92.6	−108.5
Boscartin H	−104.1	−103.6	−106.0	−104.4	−97.2	−98.8	−97.4	−94.5	−100.9	−93.4	−101.5	−99.5
Incensole	−104.3	−102.4	−101.1	−96.2	−85.0	−96.3	−90.7	−95.0	−94.0	−92.9	−93.0	−95.7
Incensole acetate	−109.9	−102.3	−96.2	−98.1	−96.6	−104.5	−96.1	−98.8	−96.2	−103.7	−91.5	−96.2
Incensole oxide	−108.6	−92.0	−93.5	−90.3	−97.4	−99.8	−94.2	−95.6	−101.4	−97.1	−103.5	−103.5
Incensole oxide acetate	−119.0	−101.1	−98.9	−96.4	−96.7	−98.7	−99.2	−100.0	−106.9	−105.5	−99.4	−100.0
Isoincensole oxide	−96.2	−87.6	−93.5	−89.9	−98.5	−97.5	−98.5	−92.7	−88.9	−87.5	−103.3	−91.2
Isoincensolol	−87.3	−91.2	−88.9	−87.9	−107.9	−90.0	−99.6	−87.8	−99.7	−91.5	−100.0	−95.2
Serratol	−90.2	−90.1	−89.9	−82.2	−90.1	−91.4	−97.6	−89.1	−103.2	−87.4	−92.7	−93.4
Verticillatriene	−74.0	−69.5	−69.0	−69.5	−81.2	−87.2	−85.5	−78.5	−77.3	−76.5	−89.9	−84.4

^a^ EcAspTA = *Escherichia coli* aspartate transaminase. SmChiB = *Serratia marcescens* chitinase. SaQacR = *Staphylococcus aureus* multidrug binding protein. HpPDF = *Helicobacter pylori* peptide deformylase. BcPhzA/B = *Burkholderia cepacia* phenazine biosynthesis protein A/B. MtBioA = *Mycobacterium tuberculosis* 7,8-diaminopelargonic acid synthase.

**Table 4:** MolDock molecular docking energies (kJ/mol) of *Boswellia* cembranoids with human immunodeficiency virus type 1 reverse transcriptase (HIV1-RT).

Ligand	1eet	2hnz	3irx	3is9	3mee	3lal	3t19
Boscartin A	−99.1	−101.7	−96.8	−93.8	−96.0	−69.4	−75.4
Boscartin B	−94.1	−98.5	−100.2	−94.7	−95.6	−72.2	−97.8
Boscartin C	−104.2	−91.5	−97.0	−101.3	−100.5	−87.8	−84.0
Boscartin D	−89.2	−100.5	−97.1	−97.1	−96.4	−78.7	−83.2
Boscartin E	−97.2	−100.6	−91.7	−97.0	−89.4	−60.8	−72.0
Boscartin F	−88.9	−93.8	−98.1	−98.1	−95.1	−88.1	−69.4
Boscartin G	−92.8	−88.7	−97.6	−95.4	−113.8	+9.3	−82.3
Boscartin H	−98.2	−90.4	−96.9	−97.1	−98.2	−86.9	−56.2
Incensole	−83.8	−101.5	−88.8	−88.3	−86.8	−84.0	−94.7
Incensole acetate	−95.6	−92.6	−97.9	−97.0	−84.6	−95.9	−91.6
Incensole oxide	−105.1	−93.2	−94.9	−98.7	−106.7	−81.7	−79.0
Incensole oxide acetate	−107.2	−84.5	−106.8	−106.2	−113.5	−87.8	−87.1
Isoincensole oxide	−97.8	−86.5	−89.4	−90.2	−98.7	−77.5	−57.7
Isoincensolol	−95.2	−85.6	−98.2	−85.4	−85.9	−70.6	−72.5
Serratol	−90.4	−87.9	−86.8	−89.3	−90.1	−56.0	−48.9
Verticillatriene	−81.6	−67.1	−78.4	−81.0	−82.5	−76.9	−25.5

**Table 5:** Protein targets with the most exothermic docking energies (*E*_dock_, kJ/mol) for *Boswellia* cneorubenoid ligands.

Ligand	PDB ^a^	*E*_dock_	Target Protein
Boscartol A	3hfj	−124.2	*Bacillus anthracis* nucleotide adenyltransferase (BaNadD)
	4kn0	−125.6	human folate receptor β (HsFRβ)
	4kn1	−127.5	human folate receptor β (HsFRβ)
		−89.7	Median docking energy
Boscartol B	3hfj	−124.2	*Bacillus anthracis* nucleotide adenyltransferase (BaNadD)
	4kn0	−127.8	human folate receptor β (HsFRβ)
	4kn1	−126.8	human folate receptor β (HsFRβ)
		−91.6	Median docking energy
Boscartol C	3bt9	−120.2	*Staphylococcus aureus* multidrug binding protein (SaQacR)
		−85.8	Median docking energy
(15*R*)-Boscartol D	2cek	−120.9	*Torpedo californica* acetylcholine esterase (TcAChE)
	3bt9	−122.7	*Staphylococcus aureus* multidrug binding protein (SaQacR)
	3lal	−125.5	HIV-1 reverse transcriptase
	3t19	−123.1	HIV-1 reverse transcriptase
		−87.6	Median docking energy
(15*S*)-Boscartol D	3lal	−120.6	HIV-1 reverse transcriptase
		−81.8	Median docking energy
Boscartol E	1s9d	−125.9	bovine guanine nucleotide exchange factor (BtGEF)
	3hjf	−135.1	*Bacillus anthracis* nucleotide adenyltransferase (BaNadD)
	4b80	−124.1	murine acetylcholinesterase (MmAChE)
	4kn0	−124.2	human folate receptor β (HsFRβ)
		−85.0	Median docking energy
Boscartol F	1s9d	−124.5	bovine guanine nucleotide exchange factor (BtGEF)
	3hfj	−123.1	*Bacillus anthracis* nucleotide adenyltransferase (BaNadD)
	4kn1	−122.3	human folate receptor β (HsFRβ)
		−84.6	Median docking energy
Boscartol G	3hfj	−125.8	*Bacillus anthracis* nucleotide adenylyltransferase (BaNadD)
		−80.3	Median docking energy
Boscartol H	1dx4	−123.6	*Drosophila melanogaster* acetylcholine esterase (DmAChE)
	3bt9	−122.0	*Staphylococcus aureus* multidrug binding protein (SaQacR)
	3hfj	−127.9	*Bacillus anthracis* nucleotide adenyltransferase (BaNadD)
	4kn0	−124.6	human folate receptor β (HsFRβ)
		−85.9	Median docking energy
Boscartol I	3hfj	−133.8	*Bacillus anthracis* nucleotide adenyltransferase (BaNadD)
	3p2v	−121.1	human aldose reductase (HsAR)
	4kn0	−127.3	human folate receptor β (HsFRβ)
		−91.4	Median docking energy
Olibanumol D	4kn1	−120.6	human folate receptor β (HsFRβ)
		−78.4	Median docking energy

**^a^** PDB: Protein Data Bank code.

**Table 6:** Protein targets with the most exothermic docking energies (*E*_dock_, kJ/mol) for *Boswellia* triterpenoid ligands.

Ligand	PDB ^a^	*E*_dock_	Target
(20*S*)-3,7-Dioxotirucalla-8,24-dien-21-oic acid	3l3m	−141.8	Human poly(ADP-ribose) polymerase-1 (HsPARP-1) (anticancer target)
	3ua9	−140.3	Human tankyrase-2 (HsTANK2) = human poly(ADP-ribose) polymerase-5b (HsPARP-5b) (antitumor target)
	3g49	−132.4	*Cavia porcellus* 11β-hydroxysteroid dehydrogenase type 1 (Cp11βHSD1) (diabetes target)
	2b03	−127.4	Porcine pancreatic phospholipase A2 (SsPLA2) (anti-inflammatory target)
		−91.3	Median docking energy
11-Ethoxy-β-boswellic acid	3i6m	−124.6	*Torpedo californica* acetylcholinesterase (TcAChE) (Alzheimer’s target)
		−63.0	Median docking energy
11-Keto-β-boswellic acid	4b84	−125.3	Murine acetylcholinesterase (MmAChE) (Alzheimer’s target)
	3i6m	−120.8	*Torpedo californica* acetylcholinesterase (TcAChE) (Alzheimer’s target)
		−59.9	Median docking energy
12-Ursen-3,11-dione	1h22	−118.8	*Torpedo californica* acetylcholinesterase (TcAChE) (Alzheimer’s target)
		−64.2	Median docking energy
12-Ursen-3,24-diol	1h22	−123.8	*Torpedo californica* acetylcholinesterase (TcAChE) (Alzheimer’s target)
		+17.4	Median docking energy
2,3-Dihydroxy-12-ursen-24-oic acid	4b84	−128.0	Murine acetylcholinesterase (MmAChE) (Alzheimer’s target)
		+16.2	Median docking energy
20,22-Epoxytirucall-24-en-3-one	3l3m	−133.5	Human poly(ADP-ribose) polymerase-1 (HsPARP-1) (anticancer target)
	3g49	−125.0	*Cavia porcellus* 11β-hydroxysteroid dehydrogenase type 1 (Cp11βHSD1) (diabetes target)
		−88.9	Median docking energy
24-Nor-3,12-ursadien-11-one	1h22	−121.0	*Torpedo californica* acetylcholinesterase (TcAChE) (Alzheimer’s target)
	4b84	−120.9	Murine acetylcholinesterase (MmAChE) (Alzheimer’s target)
		+30.9	Median docking energy
24-Nor-3,9(11),12-oleanatriene	3lz6	−112.6	*Cavia porcellus* 11β-hydroxysteroid dehydrogenase type 1 (Cp11βHSD1) (diabetes target)
		−31.2	Median docking energy
24-Nor-3,9(11),12-ursatriene	1h22	−118.4	*Torpedo californica* acetylcholinesterase (TcAChE) (Alzheimer’s target)
		+15.1	Median docking energy
3-Acetoxy-12,20(29)-lupadien-24-oic acid	1uk1	−118.4	Human poly(ADP-ribose) polymerase-1 (HsPARP-1) (anticancer target)
	3bti	−117.9	*Staphylococcus aureus* multidrug binding protein (SaQacR)
		+16.4	Median docking energy
3-Acetoxy-20(29)-lupen-24-oic acid (= 3-Acetyl lupeolic acid)	1cgl	−117.5	Human fibroblast collagenase (HsMMP-1) (arthritis target)
		+8.0	Median docking energy
3β-Acetoxy-20*S*,24*R*-dihydroxydammar-25-ene ^b^	2aba	−144.7	*Enterobacter cloacae* pentaerythritol tetranitrate reductase (EcPETNR) (antibacterial target)
	3lz6	−141.8	*Cavia porcellus* 11β-hydroxysteroid dehydrogenase type 1 (Cp11βHSD1) (diabetes target)
	1h36	−136.4	*Alicyclobacillus acidocardarius* oxidosqualene cyclase (Aa OSC) (cholesterol-lowering)
		−64.6	Median docking energy
3β-Acetoxy-20*S*,24*S*-dihydroxydammar-25-ene ^b^	2aba	−149.9	*Enterobacter cloacae* pentaerythritol tetranitrate reductase (EcPETNR) (antibacterial target)
	3g49	−139.7	*Cavia porcellus* 11β-hydroxysteroid dehydrogenase type 1 (Cp11βHSD1) (diabetes target)
	3lz6	−136.2	*Cavia porcellus* 11β-hydroxysteroid dehydrogenase type 1 (Cp11βHSD1) (diabetes target)
	3tfu	−134.5	*Mycobacterium tuberculosis* 7,8-diaminopelargonic acid synthase (MtBioA)
		−73.7	Median docking energy
3-Acetoxy-5,12-ursadien-24-oic acid	3i6m	−131.7	*Torpedo californica* acetylcholinesterase (TcAChE) (Alzheimer’s target)
		−66.7	Median docking energy
3-Acetyl-11-keto-β-boswellic acid	3i6m	−129.7	*Torpedo californica* acetylcholinesterase (TcAChE) (Alzheimer’s target)
		−70.7	Median docking energy
3-Acetyl-11α-methoxy-β-boswellic_acid	3i6m	−129.0	*Torpedo californica* acetylcholinesterase (TcAChE) (Alzheimer’s target)
		−72.2	Median docking energy
3-Acetyl-9,11-dehydro-β-boswellic_acid	3i6m	−124.0	*Torpedo californica* acetylcholinesterase (TcAChE) (Alzheimer’s target)
		−64.4	Median docking energy
3-Acetyl-α-boswellic acid	2b03	−112.7	Porcine pancreatic phospholipase A2 (SsPLA2) (anti-inflammatory target)
		−64.8	Median docking energy
3-Acetyl-β-boswellic acid	3i6m	−128.3	*Torpedo californica* acetylcholinesterase (TcAChE) (Alzheimer’s target)
		−71.5	Median docking energy
3-Hydroxy-20(29)-lupen-24-oic acid (= Lupeolic acid)	3bt9	−119.3	*Staphylococcus aureus* multidrug binding protein (SaQacR)
		−55.8	Median docking energy
3-Oxotirucalla-7,9(11),24-trien-21-oic acid	3ua9	−151.0	Human tankyrase-2 (HsTANK2) = human poly(ADP-ribose) polymerase-5b (HsPARP-5b) (antitumor target)
	3l3m	−137.6	Human poly(ADP-ribose) polymerase-1 (HsPARP-1) (anticancer target)
	3h6k	−130.9	Human 11β-hydroxysteroid dehydrogenase type 1 (11βHSD1) (diabetes target)
	1w6j	−127.5	Human oxidosqualene cyclase (HsOSC) (hypercholesterolemia target)
	3bt9	−127.1	*Staphylococcus aureus* multidrug binding protein (SaQacR)
		−96.8	Median docking energy
3β-Hydroxytirucalla-8,24-dien-21-oic acid	3ua9	−144.5	Human tankyrase-2 (HsTANK2) = human poly(ADP-ribose) polymerase-5b (HsPARP-5b) (antitumor target)
	3l3m	−133.0	Human poly(ADP-ribose) polymerase-1 (HsPARP-1) (anticancer target)
	3g49	−132.3	*Cavia porcellus* 11β-hydroxysteroid dehydrogenase type 1 (Cp11βHSD1) (diabetes target)
	2ilt	−129.1	Human 11β-hydroxysteroid-dehydrogenase (Hs11β-HSDH) (diabetes target)
	4krs	−127.7	Human tankyrase-1 (HsTANK1) (anticancer target)
	4l0i	−127.3	Human tankyrase-2 (HsTANK2) (anticancer target)
		−85.6	Median docking energy
3α-Acetoxytirucalla-7,24-dien-21-oic acid	3ua9	−147.1	Human tankyrase-2 (HsTANK2) = human poly(ADP-ribose) polymerase-5b (HsPARP-5b) (antitumor target)
	4krs	−134.1	Human tankyrase-1 (HsTANK1) (anticancer target)
	1cgl	−129.9	Human fibroblast collagenase (HsMMP-1) (arthritis target)
	4gv0	−128.4	Human poly(ADP-ribose) polymerase-1 (HsPARP-1) (anticancer target)
	4l0i	−128.4	Human tankyrase-2 (HsTANK2) (anticancer target)
	3lep	−126.8	Human aldose reductase (HsAR) (diabetes target)
		−102.1	Median docking energy
3α-Hydroxytirucalla-7,24-dien-21-oic acid	3ua9	−154.3	Human tankyrase-2 (HsTANK2) = human poly(ADP-ribose) polymerase-5b (HsPARP-5b) (antitumor target)
	3g49	−128.9	*Cavia porcellus* 11β-hydroxysteroid dehydrogenase type 1 (Cp11βHSD1) (diabetes target)
	3l3m	−126.7	Human poly(ADP-ribose) polymerase-1 (HsPARP-1) (anticancer target)
	3tfu	−126.4	*Mycobacterium tuberculosis* 7,8-diaminopelargonic acid synthase (MtBioA)
		−88.7	Median docking energy
3β-Acetoxydammar-24-ene-16β,20*R*-diol	3lz6	−149.6	*Cavia porcellus* 11β-hydroxysteroid dehydrogenase type 1 (Cp11βHSD1) (diabetes target)
	2aba	−141.1	*Enterobacter cloacae* pentaerythritol tetranitrate reductase (EcPETNR) (antibacterial target)
	1h36	−139.2	*Alicyclobacillus acidocardarius* oxidosqualene cyclase (AaOSC) (cholesterol-lowering)
	1s0x	−131.2	Human retinoic acid-related orphan receptor α (HsRORα) (may regulate lipid metabolism)
		−54.0	Median docking energy
3β-Acetoxylup-20(29)-en-11β-ol	1uk1	−121.9	Human poly(ADP-ribose) polymerase-1 (HsPARP-1) (anticancer target)
		−50.4	Median docking energy
4,23-Dihydroburic acid	1h22	−135.8	*Torpedo californica* acetylcholinesterase (TcAChE) (Alzheimer’s target)
	4b84	−127.8	Murine acetylcholinesterase (MmAChE) (Alzheimer’s target)
	1jtx	−125.1	*Staphylococcus aureus* multidrug binding protein (SaQacR)
		+11.7	Median docking energy
6,7-Epoxy-9(11)-oleanen-3-ol	4hai	−111.6	Human soluble epoxide hydrolase (HsEPHX2) (anti-inflammatory target)
		−60.7	Median docking energy
6,7-Epoxy-9(11)-oleanen-3-one	1ry0	−113.7	Human prostaglandin F synthase (HsPGFS) (hypertension target)
		−49.1	Median docking energy
9,11-Dehydro-β-boswellic acid	1qvu	−114.3	*Staphylococcus aureus* multidrug binding protein (SaQacR)
		−60.8	Median docking energy
Boscartene L	3l3m	−131.0	Human poly(ADP-ribose) polymerase-1 (HsPARP-1) (anticancer target)
		−87.6	Median docking energy
Boscartene M	3l3m	−133.7	Human poly(ADP-ribose) polymerase-1 (HsPARP-1) (anticancer target)
		−89.4	Median docking energy
Boscartene N	3ua9	−157.2	Human tankyrase-2 (HsTANK2) = human poly(ADP-ribose) polymerase-5b (HsPARP-5b) (antitumor target)
	3bt9	−136.7	*Staphylococcus aureus* multidrug binding protein (SaQacR)
	3l3m	−133.3	Human poly(ADP-ribose) polymerase-1 (HsPARP-1) (anticancer target)
	1xl5	−125.2	HIV-1 protease
		−95.1	Median docking energy
Dammarenediol II	1w6k	−135.1	Human oxidosqualene cyclase (HsOSC) (hypercholesterolemia target)
	3lz6	−134.6	*Cavia porcellus* 11β-hydroxysteroid dehydrogenase type 1 (Cp11βHSD1) (diabetes target)
	1h36	−129.2	*Alicyclobacillus acidocardarius* oxidosqualene cyclase (AaOSC) (cholesterol-lowering)
		−72.1	Median docking energy
Dammarenediol II acetate	3lz6	−142.8	*Cavia porcellus* 11β-hydroxysteroid dehydrogenase type 1 (Cp11βHSD1) (diabetes target)
	1h36	−138.9	*Alicyclobacillus acidocardarius* oxidosqualene cyclase (AaOSC) (cholesterol-lowering)
	2aba	−136.4	*Enterobacter cloacae* pentaerythritol tetranitrate reductase (EcNETNR) (antibacterial target)
	4kn0	−132.3	Human folate receptor β (HsFRβ) (anticancer target)
		−77.7	Median docking energy
Eupha-2,8,22-triene-20,24*R*-diol ^b^	3l3m	−139.8	Human poly(ADP-ribose) polymerase-1 (HsPARP-1) (anticancer target)
	3ua9	−135.3	Human tankyrase-2 (HsTANK2) = human poly(ADP-ribose) polymerase-5b (HsPARP-5b) (antitumor target)
	4dbs	−124.0	Human estrogenic 17β-hydroxysteroid dehydrogenase (17β-HSD1)
		−94.9	Median docking energy
Eupha-2,8,22-triene-20,24*S*-diol ^b^	1uk1	−137.6	Human poly(ADP-ribose) polymerase-1 (HsPARP-1) (anticancer target)
	3ua9	−135.8	Human tankyrase-2 (HsTANK2) = human poly(ADP-ribose) polymerase-5b (HsPARP-5b) (antitumor target)
	4dbs	−127.4	Human estrogenic 17β-hydroxysteroid dehydrogenase (17β-HSD1)
		−85.1	Median docking energy
Isofouquierol	3lz6	−134.1	*Cavia porcellus* 11β-hydroxysteroid dehydrogenase type 1 (Cp11βHSD1) (diabetes target)
	2aba	−131.3	*Enterobacter cloacae* pentaerythritol tetranitrate reductase (EcPETNR) (antibacterial target)
	4kn0	−130.4	Human folate receptor β (HsFRβ) (anticancer target)
		−83.2	Median docking energy
Isofouquieryl acetate	3lz6	−150.1	*Cavia porcellus* 11β-hydroxysteroid dehydrogenase type 1 (Cp11βHSD1) (diabetes target)
	2aba	−137.9	*Enterobacter cloacae* pentaerythritol tetranitrate reductase (EcPETNR) (antibacterial target)
	4kn0	−137.0	Human folate receptor β (HsFRβ) (anticancer target)
	1s0x	−135.7	Human retinoic acid-related orphan receptor α (HsRORα) (may regulate lipid metabolism)
	1h35	−135.5	*Alicyclobacillus acidocardarius* oxidosqualene cyclase (AaOSC) (cholesterol-lowering)
	2w4q	−132.7	Human zinc-binding alcohol dehydrogenase 1 (HsZADH1)
	3g49	−132.4	*Cavia porcellus* 11β-hydroxysteroid dehydrogenase type 1 (Cp11βHSD1) (diabetes target)
	2zxm	−131.0	Rat vitamin D receptor (RnVDR) (target for psoriasis)
		−79.3	Median docking energy
Lup-20(29)-ene-2α,3β-diol	1xu9	−112.5	Human 11β-hydroxysteroid dehydrogenase type 1 (11βHSD1) (diabetes target)
	3wd2	−112.4	*Serratia marcescens* chitinase B (SmChiB)
	1xl5	−112.2	HIV-1 protease
		−62.2	Median docking energy
Neoilexonol	1h22	−118.2	*Torpedo californica* acetylcholinesterase (TcAChE) (Alzheimer’s target)
		−66.7	Median docking energy
Neoilexonyl acetate	2ilt	−118.8	Human 11β-hydroxysteroid-dehydrogenase (Hs11β-HSDH) (diabetes target)
	4b84	−118.5	Murine acetylcholinesterase (MmAChE) (Alzheimer’s target)
		−74.8	Median docking energy
Nizwanone	4b84	−123.2	Murine acetylcholinesterase (MmAChE) (Alzheimer’s target)
		−63.3	Median docking energy
Ocotillyl acetate	1gsz	−144.6	*Alicyclobacillus acidocardarius* oxidosqualene cyclase (AaOSC) (cholesterol-lowering)
	2aba	−130.1	*Enterobacter cloacae* pentaerythritol tetranitrate reductase (EcPETNR) (antibacterial target)
	4jbs	−127.7	Human endoplasmic reticulum aminopeptidase 2 (HsERAP2) (immune response target)
		−70.7	Median docking energy
Olibanumol E	2ilt	−109.5	Human 11β-hydroxysteroid-dehydrogenase (Hs11β-HSDH) (diabetes target)
		−63.5	Median docking energy
Olibanumol F	1cgl	−110.1	Human fibroblast collagenase (HsMMP-1) (arthritis target)
	3bt9	−108.8	*Staphylococcus aureus* multidrug binding protein (SaQacR)
		−36.7	Median docking energy
Olibanumol G	3bt9	−117.4	*Staphylococcus aureus* multidrug binding protein (SaQacR)
		−53.0	Median docking energy
Olibanumol H	3bt9	−121.6	*Staphylococcus aureus* multidrug binding protein (SaQacR)
		−60.9	Median docking energy
Olibanumol I	3bt9	−115.7	*Staphylococcus aureus* multidrug binding protein (SaQacR)
		−63.2	Median docking energy
Olibanumol J	1rpw	−135.5	*Staphylococcus aureus* multidrug binding protein (SaQacR)
	1cr6	−133.3	Murine soluble epoxide hydrolase (MmEPHX2) (anti-inflammatory target)
	3bti	−132.6	*Staphylococcus aureus* multidrug binding protein (SaQacR)
	3gyt	−132.1	*Strongyloides stercoralis* nuclear receptor DAF-12 (SsDAF12) (antiparasitic target)
		−55.5	Median docking energy
Olibanumol J*’*	3l3m	−132.3	Human poly(ADP-ribose) polymerase-1 (HsPARP-1) (anticancer target)
	3g49	−129.0	*Cavia porcellus* 11β-hydroxysteroid dehydrogenase type 1 (Cp11βHSD1) (diabetes target)
	3w5e	−128.8	Human phosphodiesterase 4B (HsPDE4B) (anti-inflammatory target)
	3bt9	−123.4	*Staphylococcus aureus* multidrug binding protein (SaQacR)
		−74.7	Median docking energy
Olibanumol K	4b84	−122.9	Murine acetylcholinesterase (MmAChE) (Alzheimer’s target)
	3i6m	−122.1	*Torpedo californica* acetylcholinesterase (TcAChE) (Alzheimer’s target)
		−64.8	Median docking energy
Olibanumol L	1ry0	−116.2	Human prostaglandin F synthase (HsPGFS) (hypertension target)
	3g49	−111.1	*Cavia porcellus* 11β-hydroxysteroid dehydrogenase type 1 (Cp11βHSD1) (diabetes target)
		−27.6	Median docking energy
Olibanumol L*’*	4b84	−130.1	Murine acetylcholinesterase (MmAChE) (Alzheimer’s target)
		−70.7	Median docking energy
Olibanumol M	1h22	−128.4	*Torpedo californica* acetylcholinesterase (TcAChE) (Alzheimer’s target)
		−62.7	Median docking energy
Olibanumol N	4b84	−133.0	Murine acetylcholinesterase (MmAChE) (Alzheimer’s target)
		−70.9	Median docking energy
Trametenolic acid B	3ua9	−131.6	Human tankyrase-2 (HsTANK2) = human poly(ADP-ribose) polymerase-5b (HsPARP-5b) (antitumor target)
	3g49	−127.1	*Cavia porcellus* 11β-hydroxysteroid dehydrogenase type 1 (11βHSD1) (diabetes target)
	3hfb	−126.6	Human tryoptophan hydroxylase type 1 (HsTPH1) (biosynthesis of serotonin)
	1eve	−126.5	*Torpedo californica* acetylcholinesterase (TcAChE) (Alzheimer’s target)
		−74.2	Median docking energy
Urs-12-ene-3α,11α-diol	1h22	−120.9	*Torpedo californica* acetylcholinesterase (TcAChE) (Alzheimer’s target)
		−61.1	Median docking energy
Urs-12-ene-3β,11α-diol	4b84	−123.7	Murine acetylcholinesterase (MmAChE) (Alzheimer’s target)
		−59.8	Median docking energy
α-Boswellic acid	3g49	−110.0	*Cavia porcellus* 11β-hydroxysteroid dehydrogenase type 1 (11βHSD1) (diabetes target)
		−65.7	Median docking energy
α-Elemolic acid	3ua9	−152.7	Human tankyrase-2 (HsTANK2) = human poly(ADP-ribose) polymerase-5b (HsPARP-5b) (antitumor target)
	1c3s	−136.9	*Aquifex aeolicus* histone deacetylase (AaHDAC) (anticancer target)
	3g49	−128.9	*Cavia porcellus* 11β-hydroxysteroid dehydrogenase type 1 (11βHSD1) (diabetes target)
		−87.8	Median docking energy
β-Boswellic acid	3i6m	−118.1	*Torpedo californica* acetylcholinesterase (TcAChE) (Alzheimer’s target)
		+20.7	Median docking energy
β-Elemonic acid	3ua9	−147.3	Human tankyrase-2 (HsTANK2) = human poly(ADP-ribose) polymerase-5b (HsPARP-5b) (antitumor target)
	3l3m	−133.7	Human poly(ADP-ribose) polymerase-1 (HsPARP-1) (anticancer target)
	3g49	−131.9	*Cavia porcellus* 11β-hydroxysteroid dehydrogenase type 1 (11βHSD1) (diabetes target)
		−89.0	Median docking energy
δ-Boswellic acid	3g49	−106.7	*Cavia porcellus* 11β-hydroxysteroid dehydrogenase type 1 (11βHSD1) (diabetes target)
		−60.7	Median docking energy

**^a^** PDB: Protein Data Bank code. ^b^ The stereochemistry at C(24) was not experimentally determined for 3β-acetoxy-20*S*,24-dihydroxydammar-25-ene or for eupha-2,8,22-triene-20,24-diol; both diastereomers for each of these compounds were examined in this reverse docking study.

**Table 7:** MolDock docking energies (kJ/mol) of *Boswellia* triterpenoid ligands with inflammation-relevant protein targets.

Ligand	MmEPHX2	HsEPHX2	HsMMP-1	SsPLA2	HsPLA2	Hs5-LOX	Mm iNOS	HsPI3Kγ	HsIRAK4	HsERAP2	HsGSTO1	HsPDE4B
	1cr6	4hai	1cgl	2b03	1j1a	3v99	1m8d	2a5u	5t1s	4jbs	5v3q	3w5e
(20*S*)-3,7-Dioxotirucalla-8,24-dien-21-oic acid	−113.3	−114.1	−121.8	−127.4	−106.9	−103.1	−88.0	−87.7	−112.7	−107.3	−106.0	−114.0
11-Ethoxy-β-boswellic acid	−80.3	−96.3	−107.9	−100.9	−87.3	−85.8	−71.7	−82.5	−99.7	−104.5	−66.8	−101.5
11-Keto-β-boswellic acid	−75.7	−91.8	−100.7	−94.9	−85.3	−83.3	−64.2	−77.2	−89.7	−99.4	−70.7	2.8
12-Ursen-3,11-dione	−73.9	−88.8	−102.0	−102.4	−71.2	−75.8	−63.0	−74.9	−82.0	−98.6	−71.4	−99.9
12-Ursen-3,24-diol	−81.9	−84.5	−105.5	−88.7	−93.7	−77.4	−60.4	−76.9	−89.8	−96.4	−87.8	−101.1
2,3-Dihydroxy-12-ursen-24-oic acid	−78.8	−83.9	−98.9	−100.0	−91.1	−81.7	−30.0	−78.6	−91.7	−96.5	−74.8	−102.4
20,22-Epoxytirucall-24-en-3-one	−98.2	−102.5	−113.1	−111.0	−95.7	−90.2	−89.9	−82.5	−94.7	−105.1	−80.9	−99.3
24-Nor-3,12-ursadien-11-one	−84.1	−89.6	−99.9	−103.4	−95.6	−77.5	−71.8	−75.1	−64.1	−91.7	−50.1	−83.4
24-Nor-3,9(11),12-oleanatriene	−83.4	−94.7	−99.5	−99.9	−93.3	−70.8	−61.0	−75.2	−59.9	−95.3	−47.3	−90.8
24-Nor-3,9(11),12-ursatriene	−83.3	−89.4	−100.6	−99.3	−85.5	−81.4	−65.0	−75.3	−77.0	−86.3	−62.2	−91.2
3-Acetoxy-12,20(29)-lupadien-24-oic acid	−77.8	−85.2	−115.4	−87.5	−82.7	−83.7	−68.4	−93.6	−59.1	−105.0	−78.9	−97.8
3-Acetoxy-20(29)-lupen-24-oic acid (=3-Acetyl lupeolic acid)	−84.1	−83.5	−117.5	−86.7	−87.1	−82.7	−71.8	−92.9	−79.7	−104.5	−79.0	−92.8
3β-Acetoxy-20*S*,24*R*-dihydroxydammar-25-ene	−109.7	−109.3	−124.7	−118.5	−120.3	−100.8	−96.7	−94.4	−106.4	−119.1	−85.8	−116.3
3β-Acetoxy-20*S*,24*S*-dihydroxydammar-25-ene	−106.2	−109.2	−121.7	−115.6	−122.0	−100.3	−81.5	−93.9	−109.5	−126.1	−76.5	−111.0
3-Acetoxy-5,12-ursadien-24-oic acid	−92.6	−78.1	−121.1	−97.5	−81.7	−81.0	−73.3	−83.6	−70.4	−105.0	−70.2	−47.3
3-Acetyl-11-keto-β-boswellic acid	−87.3	−75.6	−107.6	−102.4	−74.0	−84.9	−67.7	−79.7	−71.9	−93.3	−71.6	−49.2
3-Acetyl-11α-methoxy-β-boswellic_acid	−86.7	−91.1	−109.3	−91.0	−75.4	−87.3	−76.1	−79.5	−90.0	−101.9	−69.5	−52.0
3-Acetyl-9,11-dehydro-β-boswellic_acid	−90.4	−80.3	−120.6	−98.5	−94.8	−82.7	−74.4	−82.7	−61.1	−103.7	−84.3	−50.3
3-Acetyl-α-boswellic acid	−79.0	−82.4	−109.7	−112.7	−97.2	−82.0	−70.5	−83.0	−83.8	−99.7	−72.7	−91.4
3-Acetyl-β-boswellic acid	−91.5	−83.1	−119.8	−99.4	−96.2	−85.1	−69.0	−79.4	−70.8	−90.2	−80.6	−91.8
3-Hydroxy-20(29)-lupen-24-oic acid (=Lupeolic acid)	−103.4	−105.0	−106.6	−82.6	−76.6	−79.2	−72.6	−81.8	−76.0	−103.4	−69.5	−87.3
3-Oxotirucalla-7,9(11),24-trien-21-oic acid	−113.1	−113.3	−123.3	−116.9	−113.5	−100.5	−86.3	−92.1	−103.6	−108.8	−99.8	−113.2
3β-Hydroxytirucalla-8,24-dien-21-oic acid	−110.8	−112.1	−118.6	−123.4	−106.5	−103.3	−97.3	−80.7	−107.8	−109.4	−94.6	−111.9
3α-Acetoxytirucalla-7,24-dien-21-oic acid	−115.5	−113.3	−129.9	−109.8	−116.2	−91.2	−95.4	−95.3	−109.7	−106.1	−91.4	−104.9
3α-Hydroxytirucalla-7,24-dien-21-oic acid	−110.8	−115.7	−115.4	−117.2	−110.1	−105.7	−84.1	−86.9	−102.8	−108.6	−82.9	−109.8
3β-Acetoxydammar-24-ene-16β,20*R*-diol	−102.8	−110.3	−125.7	−111.2	−109.3	−97.7	−97.0	−90.7	−107.9	−125.2	−79.8	−115.2
3β-Acetoxylup-20(29)-en-11β-ol	−94.6	−92.9	−109.8	−105.6	−90.8	−86.1	−87.1	−80.9	−93.2	−108.2	−74.5	−41.2
4,23-Dihydroburic acid	−101.2	−101.6	−109.8	−120.9	−92.0	−88.3	−72.0	−84.3	−102.9	−110.2	−85.3	−107.7
6,7-Epoxy-9(11)-oleanen-3-ol	−53.0	−111.6	−100.2	−106.3	−89.8	−79.6	−64.5	−67.7	−66.2	−94.9	−68.6	−87.2
6,7-Epoxy-9(11)-oleanen-3-one	−66.0	−100.9	−105.0	−107.8	−93.3	−75.1	−68.5	−70.3	−40.3	−100.6	−66.1	−106.2
9,11-Dehydro-β-boswellic acid	−75.8	−85.7	−109.8	−96.8	−93.1	−79.8	−27.4	−73.6	−89.5	−91.2	−83.4	−62.6
Boscartene L	−83.2	−110.2	−104.4	−115.7	−77.4	−100.1	−81.6	−82.0	−100.0	−108.6	−62.6	−97.7
Boscartene M	−73.8	−110.8	−114.0	−107.0	−96.7	−102.9	−86.8	−83.4	−76.8	−110.4	−83.5	−91.5
Boscartene N	−110.2	−113.7	−116.6	−126.2	−106.6	−92.9	−85.8	−87.4	−109.6	−111.8	−100.1	−118.6
Dammarenediol II	−114.1	−112.3	−126.2	−103.7	−110.8	−95.8	−77.3	−87.2	−110.3	−108.4	−101.4	−120.5
Dammarenediol II acetate	−109.5	−109.5	−113.4	−101.8	−115.7	−105.2	−92.1	−97.4	−115.2	−107.2	−77.7	−104.9
Eupha-2,8,22-triene-20,24*R*-diol	−111.9	−106.1	−112.9	−121.3	−98.2	−97.9	−80.5	−85.9	−101.0	−106.4	−92.8	−104.5
Eupha-2,8,22-triene-20,24*S*-diol	−117.2	−104.4	−113.1	−115.5	−100.1	−97.8	−82.6	−87.3	−99.4	−103.0	−94.1	−105.8
Isofouquierol	−116.4	−103.7	−118.4	−113.5	−114.9	−104.0	−96.4	−93.2	−110.4	−114.1	−96.8	−128.3
Isofouquieryl acetate	−107.6	−119.6	−123.5	−122.4	−115.8	−96.8	−103.7	−86.4	−113.4	−124.4	−85.2	−106.6
Lup-20(29)-ene-2α,3β-diol	−101.4	−98.8	−106.0	−95.5	−83.0	−71.4	−87.9	−75.4	−89.8	−107.6	−73.2	−73.6
Neoilexonol	−73.0	−87.9	−101.1	−104.8	−71.4	−74.7	−62.8	−69.5	−52.3	−92.8	−73.9	−98.0
Neoilexonyl acetate	−86.0	−86.7	−108.2	−109.9	−83.8	−79.4	−68.2	−74.3	−87.5	−95.5	−69.4	−31.7
Nizwanone	−81.2	−89.7	−108.6	−89.5	−95.3	−78.5	−60.5	−70.1	−88.3	−98.1	−90.9	−106.3
Ocotillyl acetate	−94.4	−96.1	−121.5	−105.5	−107.2	−96.7	−72.8	−84.5	−108.2	−127.7	−77.5	−97.2
Olibanumol E	−81.7	−82.4	−101.7	−98.7	−91.8	−82.2	−60.6	−79.4	−92.4	−97.9	−49.2	−99.2
Olibanumol F	−85.7	−108.9	−110.1	−86.1	−79.7	−87.6	−65.9	−86.4	−80.4	−95.0	−77.3	−46.1
Olibanumol G	−81.5	−95.3	−99.3	−82.4	−70.4	−80.0	−61.0	−77.9	−80.8	−93.2	−54.0	−72.7
Olibanumol H	−101.1	−113.9	−107.7	−102.3	−86.8	−82.6	−78.1	−88.4	−94.2	−103.8	−74.1	−55.0
Olibanumol I	−96.2	−97.4	−101.5	−93.4	−70.6	−81.9	−75.5	−78.2	−88.8	−96.9	−71.6	−79.2
Olibanumol J	−133.3	−119.3	−114.2	−112.5	−117.6	−106.1	−93.8	−88.4	−108.3	−123.8	−99.3	−112.3
Olibanumol J’	−108.2	−119.2	−112.7	−112.3	−118.8	−107.7	−84.4	−84.3	−109.2	−124.4	−84.6	−128.8
Olibanumol K	−88.8	−77.8	−110.3	−90.5	−94.9	−77.0	−64.9	−79.8	−76.2	−89.9	−78.8	−82.4
Olibanumol L	−78.1	−80.8	−109.6	−91.7	−98.1	−81.3	−67.6	−73.8	−82.4	−97.3	−64.1	−108.1
Olibanumol L’	−85.0	−74.1	−111.4	−97.1	−83.9	−84.7	−67.6	−77.4	−72.7	−92.4	−70.0	−37.9
Olibanumol M	−77.5	−89.2	−100.1	−95.5	−57.5	−83.9	−69.2	−77.1	−96.5	−93.1	−63.9	−100.0
Olibanumol N	−85.0	−76.5	−103.3	−84.5	−70.8	−86.3	−73.6	−79.7	−87.9	−96.8	−70.4	−50.1
Trametenolic acid B	−106.3	−107.3	−106.9	−107.3	−107.9	−99.7	−92.6	−84.0	−107.6	−115.0	−92.9	−111.6
Urs-12-ene-3α,11α-diol	−77.8	−78.6	−104.3	−94.2	−85.2	−84.4	−68.3	−77.7	−92.2	−90.9	−64.6	−100.1
Urs-12-ene-3β,11α-diol	−79.4	−74.8	−101.3	−108.0	−71.2	−76.9	−67.3	−69.9	−70.9	−93.8	−61.8	−114.1
α-Boswellic acid	−77.9	−87.8	−103.7	−100.2	−95.0	−80.7	−64.4	−79.4	−85.7	−94.8	−62.5	−62.0
α-Elemolic acid	−106.5	−110.5	−113.9	−124.4	−104.1	−99.7	−107.1	−84.0	−108.7	−111.1	−97.3	−110.3
β-Boswellic acid	−78.0	−87.8	−108.9	−94.2	−90.0	−82.5	−7.8	−76.7	−91.1	−94.4	−85.2	−95.5
β-Elemonic acid	−112.2	−113.8	−110.5	−123.3	−109.6	−100.4	−92.4	−86.3	−106.6	−107.1	−108.0	−112.6
β-Elemonic acid	−110.0	−114.6	−120.2	−120.4	−106.3	−99.8	−98.8	−83.5	−109.2	−109.0	−103.6	−116.2
δ-Boswellic acid	−76.4	−90.4	−101.3	−89.6	−82.5	−74.7	−69.2	−80.7	−87.2	−96.4	−64.4	−76.4
